# Supplementary material for: Single-molecule photoreaction quantitation through intraparticle-surface energy transfer (i-SET) spectroscopy
Source: Nat Commun. 2020 Aug 27;11:4297. doi: 10.1038/s41467-020-18223-z (PMC7453008; doi:10.1038/s41467-020-18223-z)
Supplement: Supplementary file 1 — Supplementary Information [file 41467_2020_18223_MOESM1_ESM.pdf]

Supplementary Information

Single-molecule photoreaction quantitation through intraparticle-surface energy transfer (i-SET) spectroscopy

Jian Zhou, Changyu Li, Denghao Li, Xiaofeng Liu, Zhao Mu, Weibo Gao, Jianrong Qiu, and Renren Deng*

Supplementary Methods

Materials. Yttrium(Ⅲ) acetate hydrate (99.9%), ytterbium(Ⅲ) acetate hydrate (99.9%), erbium(Ⅲ) acetate hydrate (99.9%), terbium(Ⅲ) acetate hydrate (99.9%), sodium hydroxide (98+%), ammonium fluoride (99.99%), sodium trifluoroacetate (98%), terbium(Ⅲ) oxide (99.9%), 1-octadecene (90%), oleic acid (90%), were purchased from Sigma-Aldrich. Ytterbium(Ⅲ) oxide (99.99%), yttrium(Ⅲ) oxide (99.99%), lutetium oxide (99.99%), and oleylamine (80%-90%) were purchased from Aladdin. Cyclohexane, acetone, anhydrous ethanol, methanol, H_2O_2 aqueous solution (AR, 30%) and dimethylformamide were purchased from Sinopharm Chemical Reagent Co., Ltd. Commercial dye molecule BDP TMR carboxylic acid (3-[4,4-Difluoro-5-(p-methoxyphenyl)-1,3-dimethyl-3a,4a-diaza-4-bora-s-indacen-2-yl]propionic acid) was purchased from Lumiprobe. All chemicals were used as received without any purification.

Preparation of upconversion nanoparticles. The core nanoparticles were synthesized by a well-established co-precipitation method[1,2]. The core-shell nanoparticles were subsequently synthesized by a layer-by-layer hot injection method[3] using the as-synthesized core nanoparticles as seeds and cubic phase $NaLnF_4$ nanocrystals as shell precursors.

Synthesis of $Ln(CF_3COO)_3$. In a typical procedure[4], Ln_2O_3 (2.5 mmol, Ln= Lu, Y, Tb, Yb) was mixed with trifluoroacetic acid (25 mmol, in 50% aqueous solution), followed by refluxed at 110 ℃ overnight to yield a clear solution. Excessive trifluoroacetate and water were then removed through evaporation at 65 °C. The as-synthesized $Ln(CF_3COO)_3$ were dissolved and titrated with deionized water in a 50 mL volumetric flask to obtain a stock solution containing 0.2 M $Ln(CF_3COO)_3$.

Synthesis of cubic-phase $NaLnF_4$ (Ln= Lu, Y, Tb, Yb) nanocrystals. Cubic-phase $NaLnF_4$ nanocrystals were synthesized following a procedure adapted from a previously reported protocol[5,6]. In a typical experimental, 10 ml solution of $Ln(CF_3COO)_3$ (0.2 M), sodium trifluoroacetate (2 mmol), oleic acid (6.34 mL) and 1-octadecene (12.78 mL) were added into a two-neck, round-bottle flask (50 mL). The mixture was heated and maintained at 110 °C under stirring for 1.5 h until complete removal of water. Subsequently, oleylamine (6.58 mL) was added to the mixture. The reactant was then heated and kept at 125 °C for 45 min under vacuum to remove residual water and oxygen. The solution was allowed to react at 300 °C for ~25 min under nitrogen atmosphere until the reaction mixture turned turbid. After that, the reaction was kept for another 5 min and then cooled to room temperature. The as-synthesized nanocrystals were precipitated by addition of ethanol, collected by centrifugation, washed with ethanol and finally re-dispersed in cyclohexane (2 mL).

Synthesis of hexagonal-phase $NaYF_4$:Yb,Tb(60,X mol%)@$NaLuF_4$ (X= 5, 10, 20, 30, 40) core-shell nanoparticles. In a typical procedure, to a 50-mL flask containing oleic acid (3 mL) and 1-octadecene (7 mL) was added a water solution (2 mL) containing a total lanthanide amount of 0.4 mmol with designed ratios. The resulting mixture was heated at 150 °C for 1 h to form lanthanide oleate complexes and then cooled to room temperature. Subsequently, a methanol solution (6 mL) containing NaOH (1 mmol) and NH_4F (1.6 mmol) was added and stirred at 50°C

for 0.5 h. After the methanol was evaporated at 100 °C, the reaction solution was then heated to 300 °C and kept for 1 h under nitrogen flow to obtain core nanoparticles. Meanwhile, a cyclohexane dispersion containing the required amount of shell precursor nanoparticles ($NaLuF_4$) was added with 1-octadecene (4 mL), followed by bubbling nitrogen to remove cyclohexane. Thereafter, the as-synthesized shell precursor nanoparticles in 1-octadecene (1 mL) were quickly injected and ripened for 12 min to yield core-shell nanoparticles. The injection of the shell precursor was repeated another three times (1 mL each) with a ripening cycle of 12 min. After the final ripening cycle, the solution was cooled to room temperature. The as-prepared nanoparticles were collected by centrifugation, washed with ethanol several times, and finally re-dispersed in cyclohexane (4 mL).

Synthesis of hexagonal-phase $NaYbF_4$:Tb(40 mol%)@$NaLuF_4$:Tb(X mol%, X= 10, 20, 30, 40, 50, 60, 70, 80 ,90, 100) core-shell nanoparticles. The procedure is identical to the synthesis of $NaYF_4$:Yb,Tb (60, X mol%)@$NaLuF_4$, except for the use of different core- and shell-precursors during the experiment.

Synthesis of hexagonal-phase $NaYF_4$:Yb,Er(18,2 mol%)@$NaYF_4$ core-shell nanoparticles. The synthesis procedure was identical to the synthesis of $NaYF_4$:Yb,Tb (60, 40 mol%)@$NaLuF_4$ nanoparticles except for the use of different lanthanide precursors. For the synthesis of $NaYF_4$:Yb,Er(18,2 mol%) core nanoparticles, 1.6 mL of $Y(CH_3COO)_3$ (0.2 M), 0.36 mL of $Yb(CH_3COO)_3$ (0.2 M), and 0.04 mL of $Er(CH_3COO)_3$ (0.2 M), with a total lanthanide amount of 0.4 mmol were added. For the core-shell nanoparticles, 1 mL of 1-octandecene solution of cubic-phase $NaYF_4$ was used as shell precursor. The procedure of injecting and ripening cycle was identical to the synthesis of $NaLuF_4$ shell described above.

Synthesis of ligand-free nanoparticles. Ligand-free nanoparticles were obtained according to a previously reported method[7]. In a typical procedure, 50 mg oleate-capped upconversion nanoparticles (UCNPs) were dispersed in a mixture of acetone (5 mL) and hydrochloric acid (0.6 mL, 12 M), followed by ultrasonication for 30 min to remove the oleic acid from the surface of UCNPs. The ligand-free nanoparticles were collected after centrifugation at 4000 rpm for 10 min, washed with acetone three times, and redispersed in methanol (1 mL).

Synthesis of BDP-decorated upcovnersion nanoparticles for ensemble characterizations. In a typical experiment, ligand-free UCNPs (1.4 μM) and BDP TMR (1-80 μM) were mixed in a methanol solution (0.4 mL) and ultrasonicated for 10 min. The mixture was then kept in the dark overnight. After that, the BDP-decorated nanoparticles were collected by centrifugation (20000 rpm, 20 min), washed twice with acetone, and finally redispersed in methanol (0.25 mL). The exact loading concentration of BDP molecules was calculated by comparing the UV-VIS absorption spectra of BDP-loaded nanoparticles to a calibration curve.

Synthesis of BDP-decorated upcovnersion nanoparticles for single-particle spectroscopic characterizations. A cyclohexane solution of oleate-capped, core-shell nanoparticles (0.5 mL 1.1 uM) was mixed with a 2.5-mL THF solution containing BDP (1-40 μM) in a 25-mL flask. The mixture was heated to reflux at 50 °C for 2 h with vigorous stirring under nitrogen protection. The

conjugates were collected by centrifugation (20000 rpm, 20 min), washed with ethanol twice and redispersed in cyclohexane (0.7 μM, 2.5 mL). The dye loading concentration can be controlled by adjusting the concentration of BDP TMR in the THF solution.

Sample preparation for single-particle TEM and SEM characterization. The TEM and SEM characterizations were carried out to prove monodispersity of BDP-UCNP conjugates. For TEM imaging, a stock solution of BDP-decorated $NaYbF_4$:Tb@$NaTbF_4$ nanoparticles was diluted to 10 pM with cyclohexane. The solution was then dropcasted on a 20-nm thick silicon nitride membrane that contains 9 windows with a size of 100 μm × 100 μm (AR010A, CleanSiN). Note that the same size is used for confocal scanning imaging. For SEM characterization, a diluted nanoparticle solution was dropped on a gold-coated silicon wafer (0.5 cm × 0.5 cm) and then air-dried. The specimen was sputtered with Au to form an ultrathin coating to improve conductivity and contrast before being characterized by a field-emission scanning electron microscope (SU-8010).

Sample preparation of BDP-decorated UCNPs for single-nanoparticle microscopic imaging. In a typical procedure, the nanoparticle solution in cyclohexane was diluted to 2.5 pM. The diluted solution (20 uL) was then dropcasted onto a clean cover-glass (2 cm × 2 cm) and carefully rinsed using 20 uL cyclohexane. After cyclohexane evaporation, the as-prepared samples were imaged immediately under a confocal microscope.

Sample preparation of BDP-H_2O_2 decorated UCNPs for single-particle spectroscopic characterization. The procedure was identical to BDP-decorated UCNPs except using H_2O_2-cyclohexane solution to dilute. In a typical procedure, a 100-μL solution of H_2O_2 was added into 1.5-mL cyclohexane and then vortexed for 10 s. Let stand for 5 min and extract the upper cyclohexane layer to be used for dilution of BDP-decorated UCNPs. The concentration of H_2O_2 can be determined by adding different amounts of H_2O_2-cyclohexane solution while diluting solutions.

Single-nanoparticle microscopy. Single-nanoparticle optical characterization was conducted on a home built confocal microscope optical stage with a Olympus 100X NA 1.30 oil objective and a 980 nm single mode fiber laser. A data acquisition code written in Matlab to form the confocal scanning iamge, and the sample was mounted on a high precision piezoelectric stage with 100 μm × 100 μm scanning area (Physik Instrumente, P-5613CD). Photoluminescence was recorded by the photon counting module (Excelitas SPCM-AQRH-14-FC34229), and emission spectrum were recorded on the spectrometer (Princeton Instruments, ProEM) equipped with a CCD camera (eXcelon3).

Characterization of UCNPs. X-ray diffraction (XDR) data were recorded on a LabX XRD-6000 with an ADDS wide-angle X-ray powder diffractometer (Cu Kα radiation, λ=1.54184 Å). Transmission electron microscopy (TEM) images were obtained from a JEM-2100F transmission electron microscope (JEOL) operating at an acceleration voltage of 200 kV. Energy-dispersive X-ray spectroscopy (EDS) was carried out on an FEI Tecnai G2 F20 S-TWIN transmission electron microscope operated at an acceleration voltage of 200 kV. UV-vis absorption spectroscopic

measurements were performed by a UV-vis spectrophotometer (UV-2600 Shimadzu). Luminescence spectra were measured by a fluorescence spectrometer (FLSP920, Edinburgh) equipped with a continuous-wave diode laser (980nm). The luminescence decay curves were measured by a phosphorescence lifetime spectrometer (FSP 920, Edinburgh) equipped with a TTL-mode modulated, 980-nm laser diode as the pulse-excitation source. For luminescence decay measurements, the effective lifetimes were determined by

$$\tau_{eff} = \frac{1}{I_0} \int_0^{\infty} I(t) dt$$

where I_0 and $I(t)$ represent the maximum luminescence intensity and luminescence intensity at time t after cut-off of the excitation light, respectively.

Supplementary Note 1

Estimation of average loading number of BDP per nanoparticle

The average statistical BDP loading number per nanoparticle (μ) can be estimated by the equation

$$\mu = \frac{\text{BDP molar concentration}}{\text{UCNP molar concentration}}$$

Here, we take the $NaYbF_4$:Tb(40 mol%)@$NaTbF_4$ core-shell nanoparticle as an typical example to calculate the loading concentration of BDP per nanoparticle.

Calculation of UCNPs molar concentration: In order to estimate the molar concentration of the nanoparticle, we assume that core-shell nanoparticle is a sphere. A hexagonal $NaLnF_4$ unit cell contains 1.5 Na atom, 1.5 Ln atom and 6 F atom, which can be expressed as $Na_{1.5}Ln_{1.5}F_6$. The size of a single nanoparticle can be obtained from TEM images. For $NaYbF_4$:Tb(40 mol%)@$NaTbF_4$ core-shell nanoparticle, the radius of core and core-shell are R_c=7.25 nm and R_{cs}=11.75 nm, respectively. Therefore, the volume ration of shell to core can be calculated as 3.257 in terms of

$$\frac{V_{shell}}{V_{core}} = \left(\frac{R_{cs}}{R_c}\right)^3 - 1.$$

Then, the single UNCP composition can be redeveloped as unit cell form, which is

$$Na_{1.5}Yb_{(\frac{0.6}{1+3.257})\times 1.5}Tb_{(\frac{0.4+3.257}{1+3.257})\times 1.5}F_6 = Na_{1.5}Yb_{0.2114}Tb_{1.2886}F_6$$

Herein, the relative molecular mass of $Na_{1.5}Yb_{0.2114}Tb_{1.2886}F_6$ can be calculated as

$$M_{cell} = \frac{23\times 1.5+173\times 0.2114+159\times 1.2886+19\times 6}{N_A}$$

N_A is Avogadro constant. In addition, the volume of a $NaLnF_4$ unit cell can be obtained from JCPDS database, which is $V_{cell} = 1.05\times 10^{-22}\ cm^3$, so the density of the $NaYbF_4$:Tb(40 mol%)@$NaTbF_4$ core-shell nanoparticles can be calculated by

$$\rho = \frac{M_{cell}}{V_{cell}} = 6.169\ g\ cm^{-3}$$

The mass of a single core-shell nanoparticle can be obtained by

$$m_0 = \rho\times V_{UCNP} = \rho\times\frac{4}{3}\pi R_{CS}^3 = 4.192\times 10^{-20}\ mg$$

To measure the mass concentration of UCNPs in the stock solution, UCNPs solution (V_1= 50 μL) was dropcasted on a tared coverslip. After solvent evaporation, the mass of the nanoparticle was weighted. In the typical case, we get m_1=1.8 mg. Herein, the molar concentration of the $NaYbF_4$:Tb(40 mol%)@$NaTbF_4$ nanoparticles in stock solution can be calculated by

$$c_{UCNP} = \frac{m_1}{m_0 N_A V_1} = 1.426\ \mu M.$$

Calculation of loading concentration of BDP per nanoparticle: After conjugation of BDP with the nanoparticles, the absorbance of the BDP-nanoparticle conjugates at 542.5 nm was measured. The exact loading concentration of BDP on upconversion nanoparticles was calculated by comparing the measured absorbance to a calibration curve function (Supplementary Figure 12).

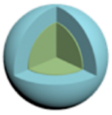

R_c=7.25 nm

R_{cs}=11.75 nm

$\frac{V_{shell}}{V_{core}} = \left(\frac{R_{cs}}{R_c}\right)^3 - 1$=3.257

Stock solution of $NaYb_{0.6}Tb_{0.4}F_4$:Tb@$NaTbF_4$

Average BDP loading number per nanoparticle

$$\mu = \frac{\textbf{BDP loading molar concentration } (c_{BDP})}{\textbf{UCNP molar concentration } (c_{UCNP})}$$

mass of single UCNP (m_0)

large unit cell: $Na_{1.5}Yb_{(\frac{0.6}{1+3.257})\times1.5}Tb_{(\frac{0.4+3.257}{1+3.257})\times1.5}F_6$

$=Na_{1.5}Yb_{0.2114}Tb_{1.2886}F_6$ (JCPDS, $Na_{1.5}Ln_{1.5}F_6$)

$$M_{cell} = \frac{23\times1.5 + 173\times0.2114 + 159\times1.2886 + 19\times6}{N_A}$$

$V_{cell} = 1.05\times10^{-22}$ cm^3 (JCPDS)

$$\rho = \frac{M_{cell}}{Vcell} = 6.169\ g/cm^3$$

$$m_0 = \rho\times V_{UCNP} = \rho\times\frac{4}{3}\pi R_{CS}^3 = 4.192\times10^{-20}\ mg$$

mass of sample UCNP (m_1)

V_1= 50 µL

m_1=1.8 mg

evaporate to weigh residue

Absorbance of UCNP-BDP

compared sample absorbance with calibration curve function

Fig. S10

UCNP molar concentration:

$$c_{UCNP} = \frac{m_1}{m_0 N_A V_1} = 1.426\ \mu M$$

BDP loading molar concentration

c_{BDP}

$$\mu = \frac{c_{BDP}}{c_{UCNP}}$$

Supplementary Note 2

Simulations on the energy transfer process

Simulation of direct energy transfer from low doping $NaYF_4$:Yb,Er(18,2 mol%)@$NaYF_4$ nanoparticles to BDP molecules

For energy transfer from upconverison nanoparticles to dye molecules, we consider that each activator is an individual energy center and acts as an energy donor. Therefore, the energy transfer is a sum of interactions of every activator to all of the dye acceptors located on nanoparticle surfaces. When $NaYF_4$:Yb,Er(18,2 mol%)@$NaYF_4$ core-shell nanoparticles are used to transfer energy to BDP molecules, the energy donors are 2% of Er^{3+} ions homogenously distributed in the core region. We assume that BDP dye molecules are randomly located on nanoparticle surfaces. In this case, we consider only the direct interaction between Er^{3+} and BDP and without Er^{3+}-Er^{3+} interaction, because of the low doping concentration of the activators. Therefore, the luminescence intensity decay process of the nanoparticle can be expressed as follows:

$$I(t) = I(0)\exp(-k_0 t - k_{DA} t) \quad \text{Eq. 1}$$

where I(t) is the emission intensity of the nanoparticle energy donor recorded at time t, k_D is the radiative rate of donor emission and k_{DA} is energy transfer rate from donor to acceptor, respectively. Considering FRET for direct energy transfer process from the low doping nanoparticle to BDP, $k_{DA}=C_{DA}r^{-6}$, where C_{DA} is the parameter relating to donor-acceptor interaction, r is the distance between donor and acceptor, and $k_0=1/\tau_0$ (τ_0 is the radiative lifetime of energy donor in the absence of dye acceptor).

We assume that the core-shell nanoparticle is a sphere. Herein, the distance distribution of the energy donors and acceptors follows a probability function P(r)[8], then $P(r)e^{-kt}dr$ can be defined as the luminescence probability of donors locating in the region of [r, r+dr] away from the acceptor recorded at time t. Considering that there are n acceptors conjugated on nanoparticle surfaces, Eq. S1 can be further written as

$$I(t) = I(0)\int\int ... \int \prod_{i=0}^{n} \{P(r_i)\exp[-k(r_i)t]\} dr_0 dr_1 ... dr_n \quad \text{Eq. 2}$$

which can be rewritten as

$$I(t) = I(0)Q(t)^n \exp(-t/t_0) \quad \text{Eq. 3}$$

where

$$Q(t) = \int P(r)\exp[-k(r)t]dr \quad \text{Eq. 4}$$

Furthermore, we assume that the average number of acceptor molecules attached to each nanoparticle obeys Poisson distribution[9] and **n** particles are considered in our simulation. Then the total luminescence intensity can be described by the following equation

$$I^{tot}(t)=\sum_{n=0}^{\infty}I_n(t)=I(0)\sum_{n=0}^{\infty}\frac{\mu^n\exp(-\mu)}{n!}Q(t)^n\exp(-t/t_0)$$

$$=I_0\exp(-\mu)\exp(-t/t_0)\sum_{n=0}^{\infty}\frac{(Q\mu)^n}{n!} \quad \text{Eq. 5}$$

Moreover, we can get that $\sum_{n=0}^{\infty}\frac{(Qu)^n}{n!}$ is Taylor's expansion item of exp(-Qμ). Therefore, Eq. 5 can be written as

$$I^{\text{tot}}(t)=I(0)\exp(-t/t_0)\exp\{-\mu[1-Q(t)]\} \quad \text{Eq. 6}$$

where μ is the average number of acceptor molecules attaching to each nanoparticle. or the expression of the direct energy transfer efficiency (Eff) by

$$Eff=1-\phi_{DA}/\phi_D=1-\frac{1}{t_0}\int_0^{\infty}\exp\{-t/t_0-\mu[1-Q(t)]\}dt \quad \text{Eq. 7}$$

For direct energy transfer model, we introduce a distance distribution function

$$P(r)=(\frac{3r}{4R*R_c})[1-(\frac{r-R}{R_c})^2] \qquad r\in[R-R_c,R+R_c]\ldots \quad \text{Eq. 8}$$

where R_c and R are the core and core-shell radius of the nanoparticles, respectively. By combining Eq. 4, 7, 8, the energy transfer efficiency *Eff* can be expressed by

$$Eff=1-\frac{1}{t_D}\int_0^{\infty}\exp[-t/t_D-\mu(1-\int_{R-R_c}^{R+R_c}(\frac{3r}{4R*R_c})[1-(\frac{r-R}{R_c})^2]\exp(-C_{DA}r^{-6}t)dr)]dt \quad \text{Eq. 9}$$

For Eq. 6, we assume 1-Q(t) = *k*t, hence the theoretical simulation curve can be plotted as the blue line, shown in Fig. 2d in the main text. Moreover, the curve fitting shows that the entire energy transfer rate from the Er^{3+}-doped nanoparticle to BDP is k≈80 s^{-1}. The whole process is schematically illustrated in Supplementary Figure 23.

Simulation of i-SET energy transfer from $NaYbF_4$:Tb(40 mol%)@$NaTbF_4$ nanoparticles to BDP molecules.

In our simulation, the i-SET process is built on high contents of active dopants in core-shell nanoparticles. These active dopants contribute significantly to energy transfer to the surface with the assistance of fast donor-donor energy migration. Compared with the direct energy transfer model that neglects the effect of energy migration among donors, the derivation of k_{DA} should consider additional donor interactions. Therefore, an average k_{DA} in the form of $\bar{k}_{DA}$ was approximately derived based on an energy migration and hopping model developed by Burshtein *et al*[10,11]. Accordingly, we derive

$$\bar{k}_{\mathrm{DA}} = \frac{1}{\tau_1^{\,2}}\int_0^{\infty} \mu[1-Q(t)]\exp(-t/\tau_1)dt = \int \mu P(r)\frac{k(r)}{1+k(r)\tau_1}dr \qquad \text{Eq. 10}$$

where k(r) is the same constant as that in Eq. 4, τ_1 denotes the average hopping time of energy migration. Similarly, we can deduce the energy transfer efficiency of the i-SET from Eq. 1

$$Eff = 1-\frac{1}{t_0}\int_0^{\infty} \exp(-t/t_0 - \bar{k}_{\mathrm{DA}}t)dt \qquad \text{Eq. 11}$$

The energy transfer is considered as Dexter's energy transfer for donor-donor and dipole-diploe interaction for donor-acceptor respectively. We suppose Tb^{3+} ions are randomly distributed in the whole particle, thus P(r) can be expressed by[8]

$$P(r) = (3r^2/4R^4)[2R-r] \qquad r \in [0,2R] \qquad \text{Eq. 12}$$

R is the radius of the particle. Furthermore, we assume that exchange interaction (Dexter`s energy transfer) dominates the energy transfer between the donors (Tb^{3+}). Herein, $1/\tau_1$ can be obtained as

$$1/\tau_1 = 2C_{DD}\exp(-1.563 N_D^{-1/3} L_{DD}^{-1}) \qquad \text{Eq. 13}$$

$$k(r) = C_{DA} r^{-6} \qquad \text{Eq. 14}$$

where C_{DD}, C_{DA} and L_{DD} are parameters denoting donor-donor interaction, donor-acceptor interaction and the spatial-overlapping degree of donor-donor wave functions. Combining Eqs. S10, S12, S13, S14, $\bar{k}_{\mathrm{DA}}$ can be written as follow:

$$\bar{k}_{\mathrm{DA}} = \mu C_{DA}\int_0^{2R} \frac{P(r)r^{-6}}{1+C_{DA}r^{-6}/2C_{DD}\exp(-1.563N_D^{-1/3}L_{DD}^{-1})}dr \qquad \text{Eq.15}$$

As illustrated in Eq. 15, N_D and μ are the only variables for any given donor-acceptor pairs. To verify the nature of donor-donor and donor-acceptor interactions in the i-SET process, we need to fit the Eq. 15 to the experimental results as a function of N_D (while keeping the particle radium R and μ as constants). Hence, we conducted measurements for a series of $NaYbF_4$:40% Tb@$NaLuF_4$:X% Tb (X=10, 30, 40, 60, 70, 100) core-shell nanoparticles with diameter 23.64 ± 1.04 nm and BDP loading concentration of μ≈3.5. The upconversion luminescence lifetime of Tb^{3+} at 547 nm was measured for these nanoparticles. The experimental k_{DA} and *Eff* values can be obtained as

$$k_{DA} = \frac{1}{\tau_{DA}} - \frac{1}{\tau_D} \qquad \text{Eq. 16}$$

$$Eff = 1 - \frac{\tau_{DA}}{\tau_D} \qquad \text{Eq. 17}$$

where τ_{DA} and τ_D are the Tb^{3+} (donor) lifetime at 547 nm with and without BDP, respectively.

As the experimental results of $\bar{k}_{DA}$ match with Eq. S15 approximately, we can derive best-fit parameters as follows: L_{DD}=0.03nm, $C_{DA}=3.03\times10^{-50}m^6s^{-1}$, $C_{DD}=2.47\times10^{17}s^{-1}$. Combining Eq. S11 and S15, the energy transfer efficiency *Eff* can be expressed as

$$Eff = 1 - \frac{1}{t_D}\int_0^{\infty} \exp[-t/t_D - \mu C_{DA} t \int_0^{2R} \frac{(3r^2/4R^4)[2R-r]r^{-6}}{1 + C_{DA} r^{-6} / 2C_{DD} \exp(-1.563 N_D^{-1/3} L_{DD}^{-1})} dr] dt$$

Eq. 18

By taking the above-fitted parameters into the Eq.18, we can derive energy transfer efficiency as a function of the number of BDP molecules per nanoparticle (μ) or particle radius (R) (see Fig. 2d black line). Since we have determined that donor-donor energy migration is through exchange interaction, then the migration rate can be calculated by the following equation

$$k(r) = C_{DD} \exp(-2r / L_{DD})$$

Eq. 19

By taking the fitted parameters into Eq. 19 and Eq. 14, the energy transfer rate of Tb-Tb and Tb-BDP can be calculated.

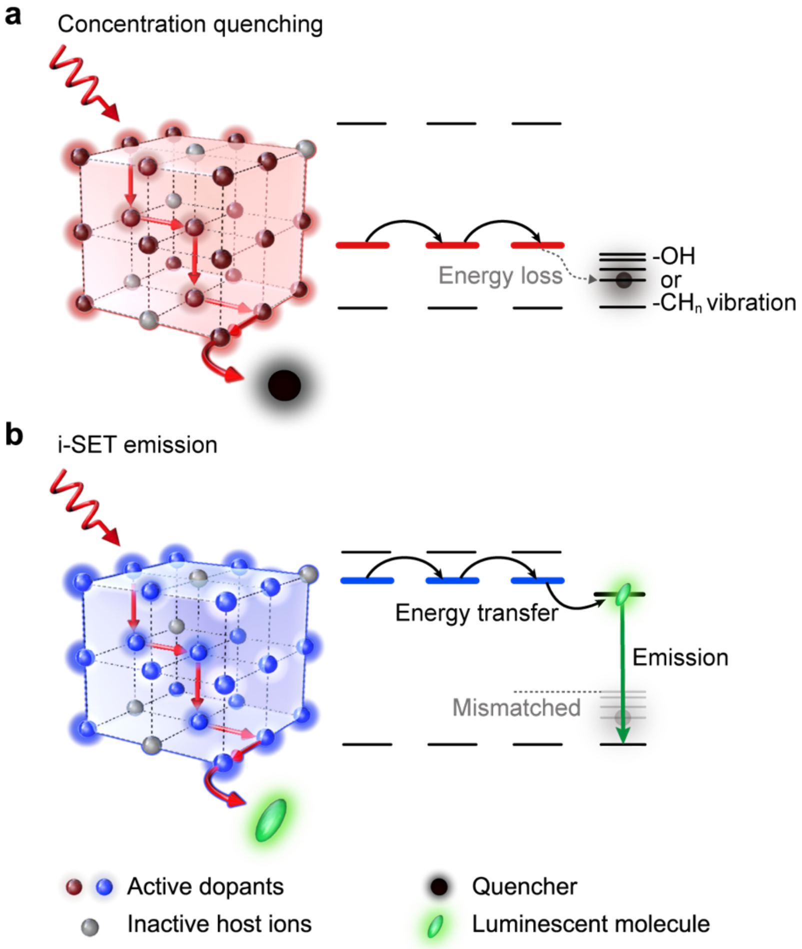

Supplementary Figure 1. Schematic illustration showing the energy transfer pathways in different types of activator sublattice. **a,** Scheme and energy transfer diagram represent the concentration quenching in a highly doped sublattice having activators with lower intermediary energy states. Long-distance energy migration through the lower intermediary energy states takes excitation energy to the vibrations of surface anchored molecules or defects. **b,** Scheme and energy transfer diagram show the i-SET achieved by using activators with only higher excitation energy levels. In this case, energy migration to the surface quenchers is unfavorable owing to the energy mismatch.

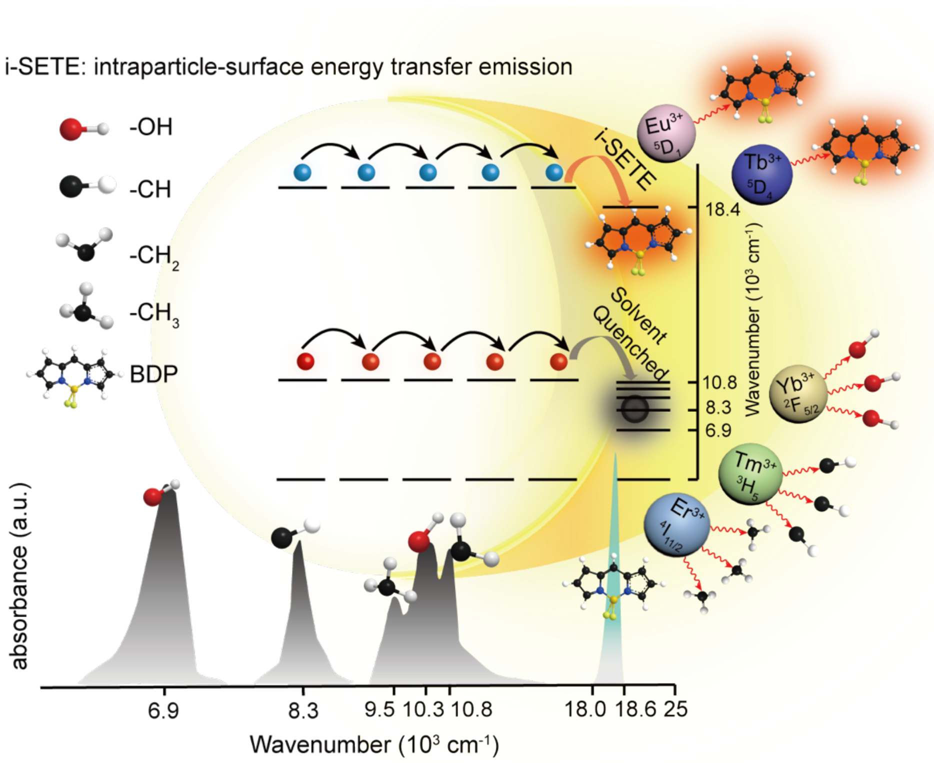

Supplementary Figure 2. Schematic illustration of energy transfer pathways of different lanthanides through either surface solvent quenching or i-SET emission. Inset data show the overtone bands absorption of vibration modes (-OH, -CH, -CH_2) measured from solvents of water, methanol, ethanol and cyclohexane.

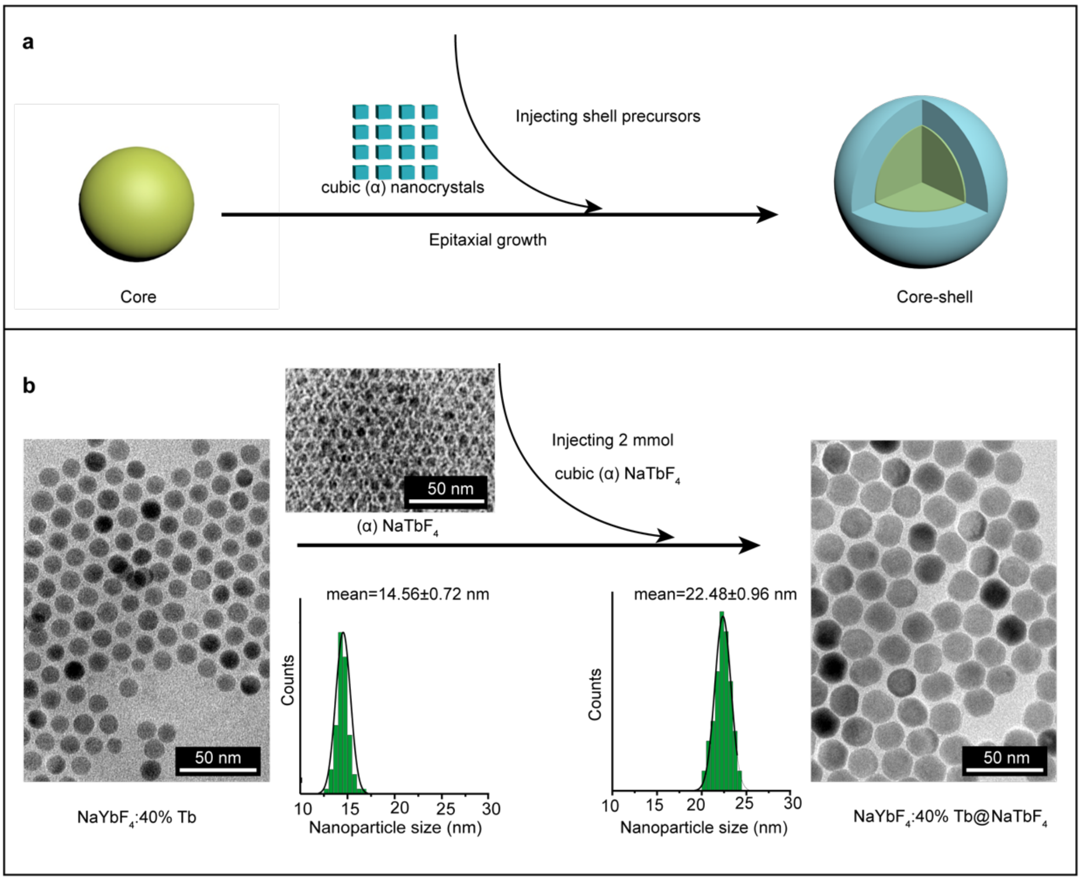

Supplementary Figure 3. a, Schematic design for the synthesis of core-shell upconversion nanoparticles. **b,** Typical TEM images and corresponding size distributions of $NaYbF_4$:Tb(40 mol%) core nanoparticles (left), and $NaYbF_4$:Tb(40 mol%)@$NaTbF_4$ core-shell nanoparticles (right), respectively. Inserted shows a typical TEM image of cubic-phase $NaTbF_4$ shell precursor. The size distributions of the nanoparticles were calculated by counting > 200 particles recorded from TEM images.

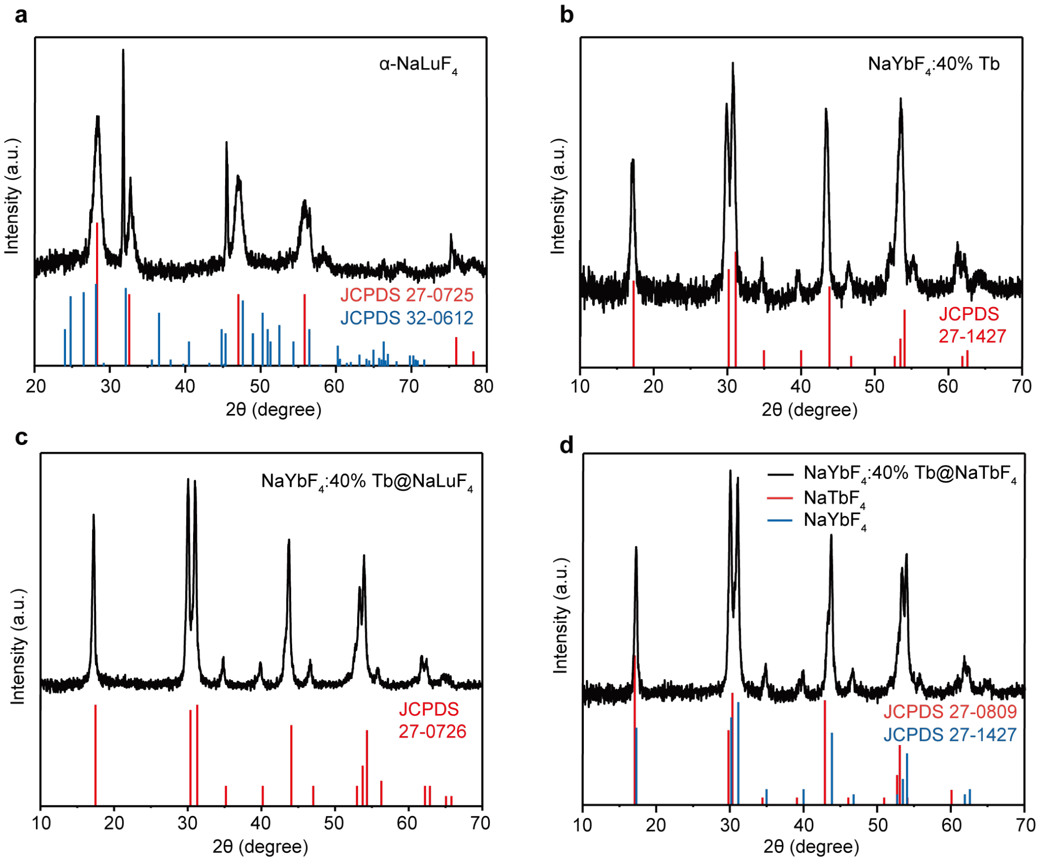

Supplementary Figure 4. X-ray powder diffraction patterns of **a,** $NaLuF_4$ shell precursors, **b,** $NaYbF_4$:Tb(40 mol%) core nanoparticles, **c,** $NaYbF_4$:Tb(40 mol%)@$NaLuF_4$ core-shell nanoparticles, and **d,** $NaYbF_4$:Tb(40 mol%)@$NaTbF_4$ core-shell nanoparticles. The $NaLuF_4$ shell precursors can be well indexed to cubic-phase $NaLuF_4$ crystal structure, while the rest of the nanoparticles are all pure hexagonal phases.

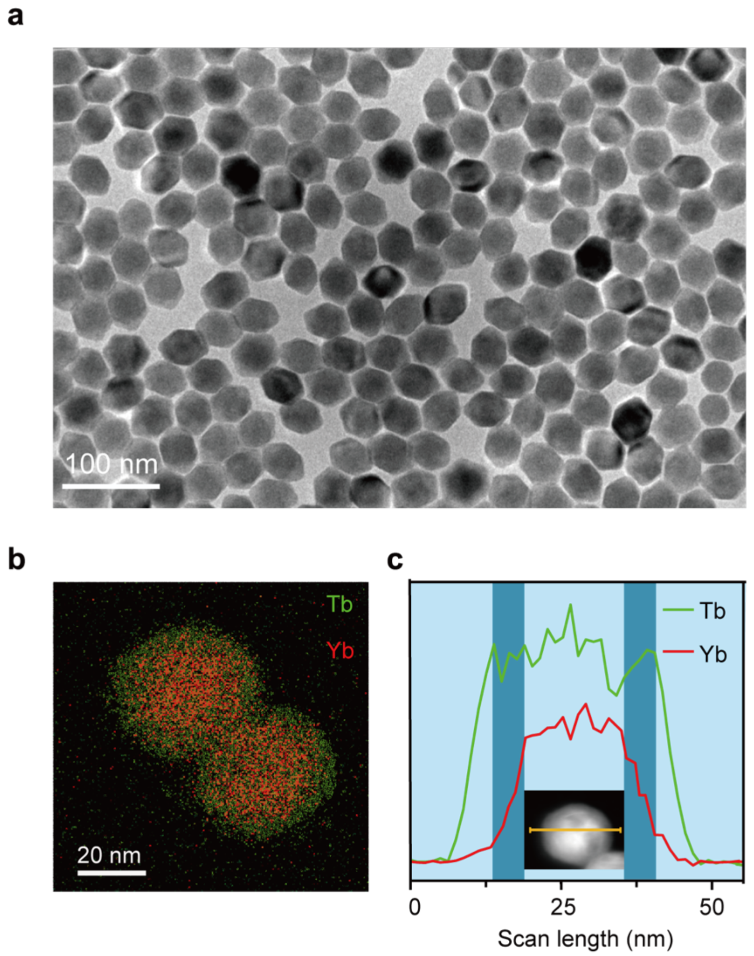

Supplementary Figure 5. a, Typical low-resolution TEM micrograph. **b,** In situ EDS elemental mapping. **c,** EDS line scan conducted with STEM imaging of the as-synthesized $NaYbF_4$:Tb(40 mol%)@$NaTbF_4$ core-shell nanoparticles.

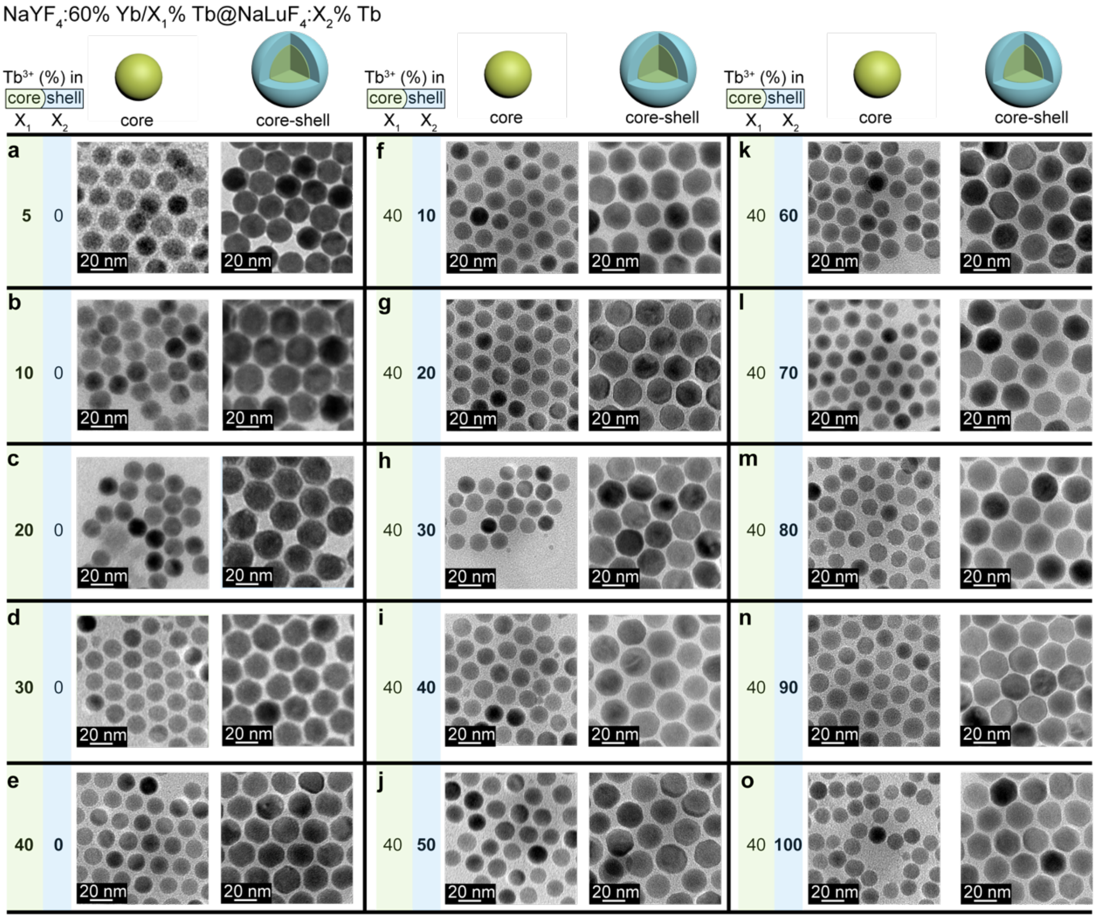

Supplementary Figure 6. TEM characterizations of **a-e,** $NaYF_4$:Yb,Tb(60, X_1 mol%)@$NaLuF_4$ (X_1=5, 10, 20, 30, 40) and **f-o,** $NaYbF_4$:Tb(40 mol%)@$NaLuF_4$:Tb(X_2 mol%, X_2=10, 20, 30, 40, 50, 60, 70, 80, 90, 100) nanoparticles. Note that the size of core and core-shell nanoparticles is similar in different Tb^{3+} doping UCNPs. All of the core nanoparticles were controlled to have a similar diameter of ~14.5 nm, which ensures the subsequently synthesized core-shell nanoparticles have a similar size of ~23.5 nm despite containing different amounts of Tb^{3+}.

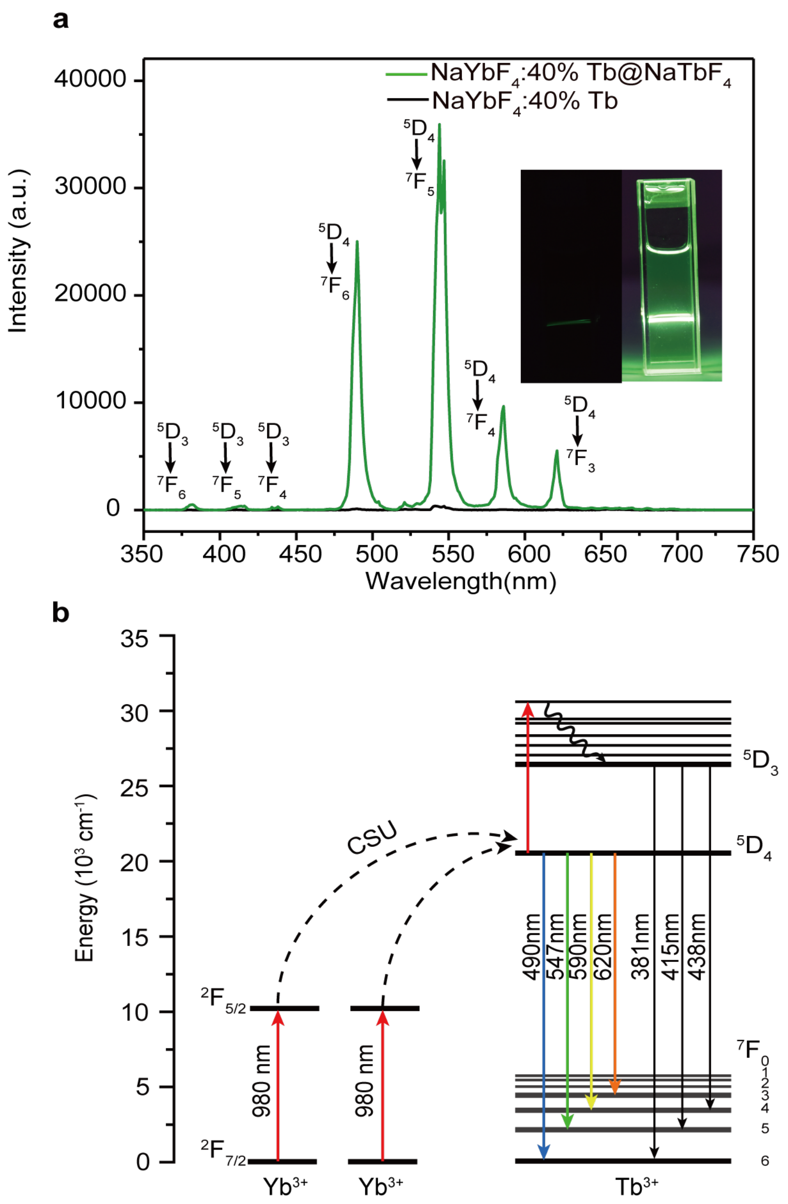

Supplementary Figure 7. **a,** Upconversion luminescence spectra of $NaYbF_4$:Tb(40 mol%) core nanoparticles and $NaYbF_4$:Tb(40 mol%)@$NaTbF_4$ core-shell nanoparticles under 980 nm excitation at 100 W/cm^2. Inset: the digital luminescence photographs showing corresponding nanoparticle colloidal. **b,** Proposed upconversion mechanism in the $NaYbF_4$:Tb(40 mol%) nanoparticles.

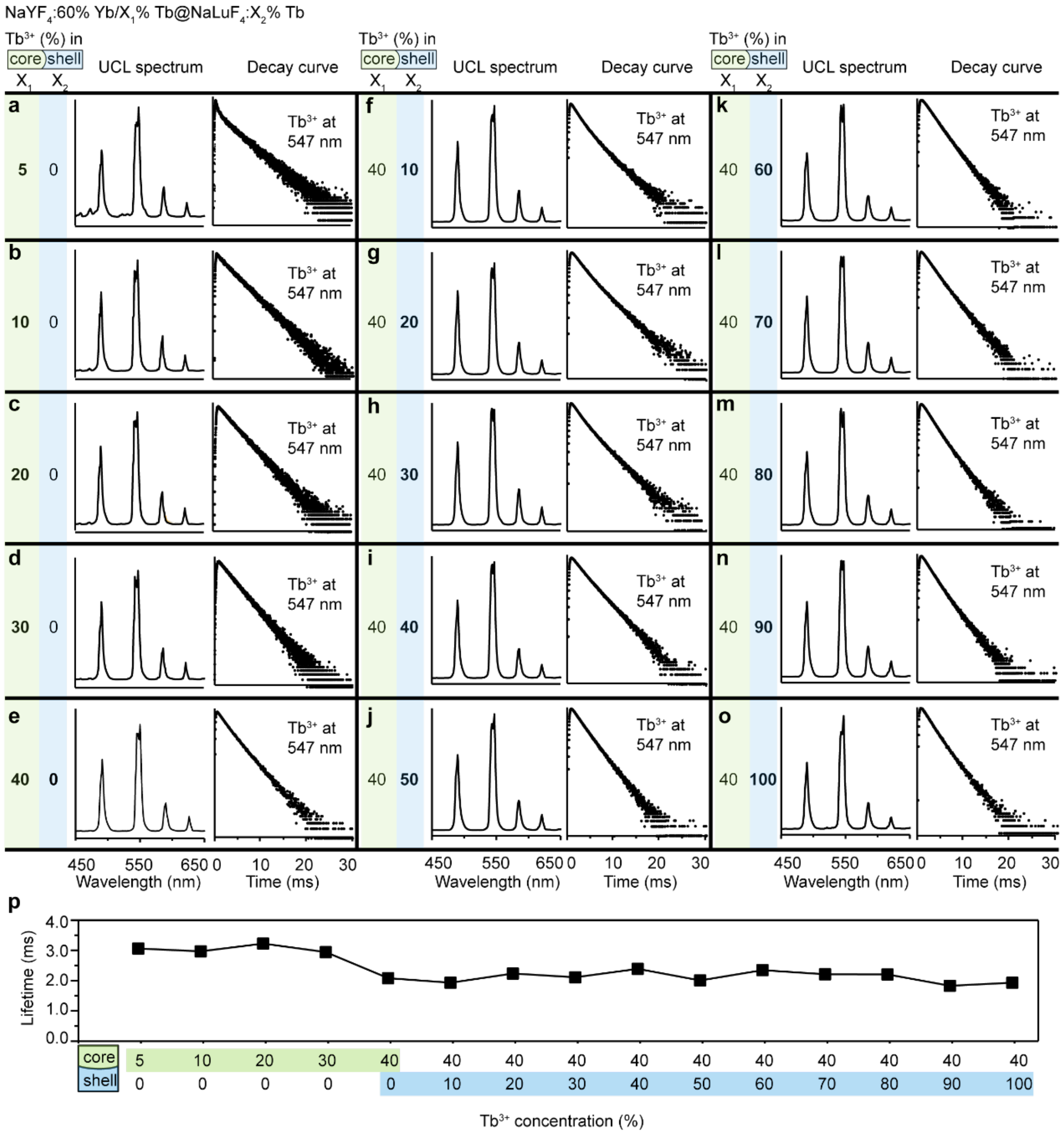

Supplementary Figure 8. Upconversion emission spectra and luminescence decay curves of **a-e**, $NaYF_4$:Yb,Tb(60, X_1 mol%)@$NaLuF_4$ (X_1=5, 10, 20, 30, 40) and **f-o,** $NaYbF_4$:Tb(40 mol%)@$NaLuF_4$:Tb(X_2 mol%, X_2=10, 20, 30, 40, 50, 60, 70, 80, 90, 100) nanoparticles. **p,** Tb^{3+} content-dependent luminescence lifetime profiles, recorded at 547 nm for the $NaYF_4$:Yb,Tb(60, X_1 mol%)@$NaLuF_4$:Tb(X_2 mol%) core-shell nanoparticles.

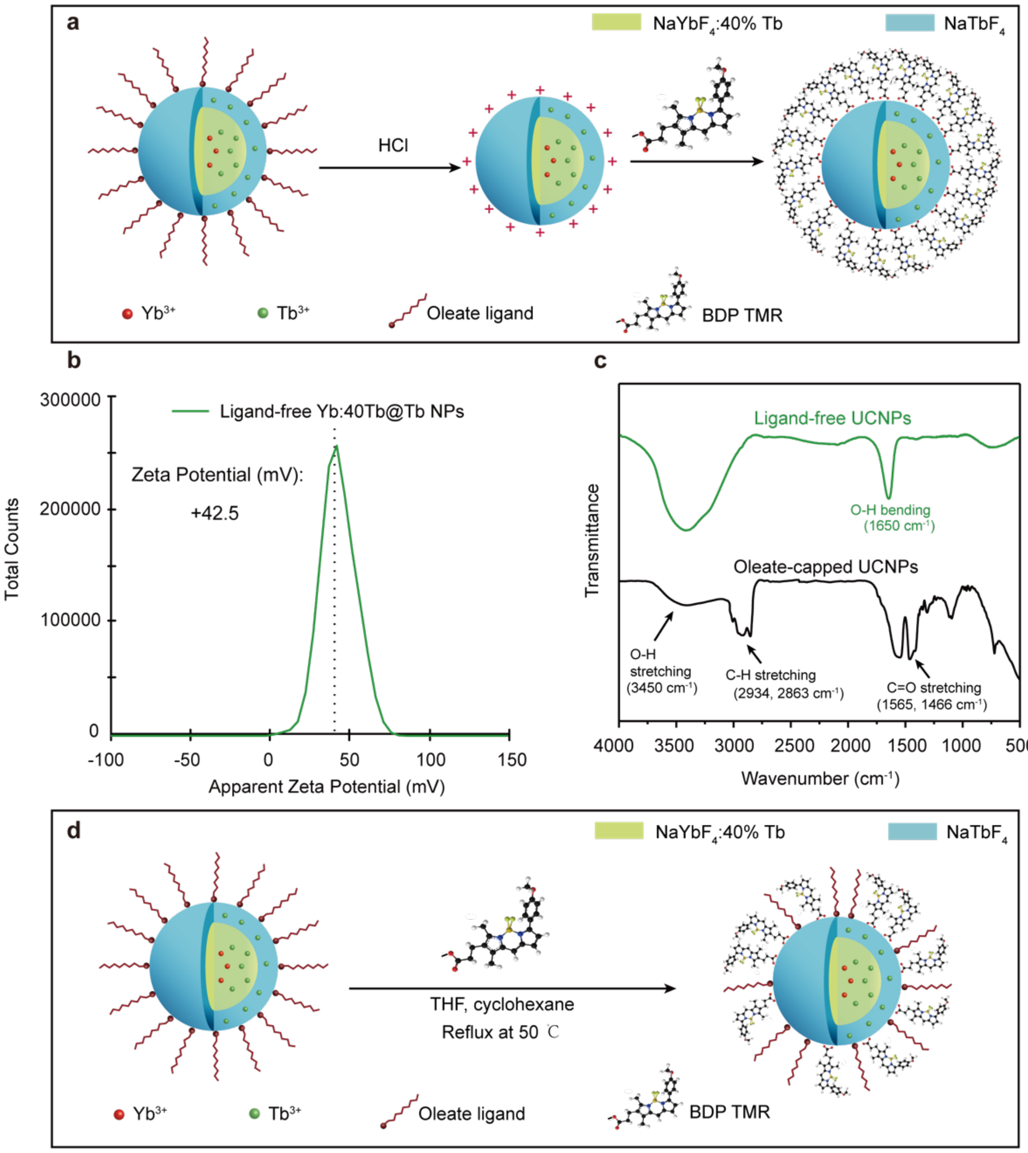

Supplementary Figure 9. Two different methods to modify upconversion nanoparticles with BDP molecules. **a,** Schematic illustration showing preparation of BDP-conjugated nanoparticles by a two-step ligand exchange method. **b,** Zeta potential measurements of ligand-free $NaYbF_4$:40% Tb@$NaTbF_4$ nanoparticles in methanol solution indicating the nanoparticles are strongly positively charged. **c,** FTIR spectra of oleate-capped and ligand-free upconversion nanoparticles, respectively. **d,** Schematic illustration showing preparation of BDP modified nanoparticles by a direct ligand exchange method.

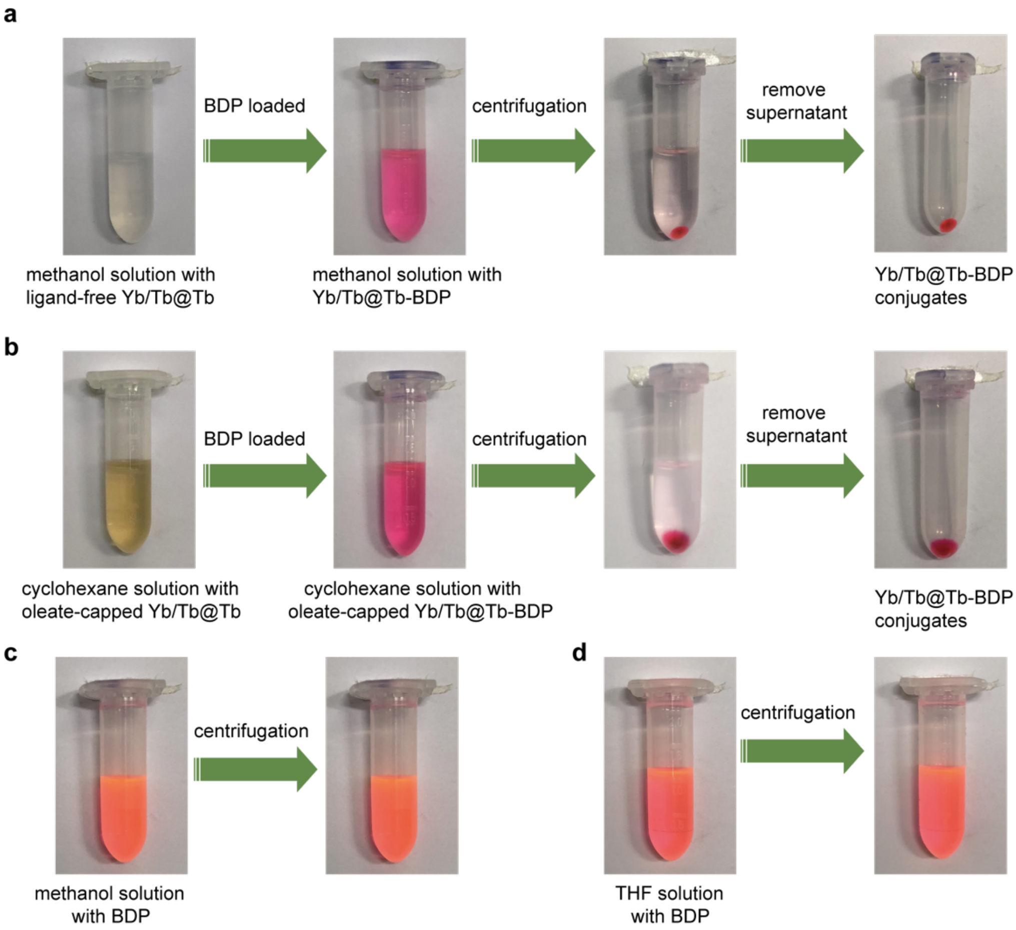

Supplementary Figure 10. The photographs of BDP loading process. **a,** Digital photos showing the loading process of BDP to ligand-free $NaYbF_4$:Tb@$NaTbF_4$ nanoparticles. **b,** Digital photos showing the loading process of BDP to oleate-capped $NaYbF_4$:Tb@$NaTbF_4$ nanoparticles. **c,** Photographs of BDP methanol solution before and after centrifuging. **d,** Photographs of BDP THF solution before and after centrifuging. The obtained colorless supernatants in nanoparticle-BDP conjugates after centrifugation indicate the successful loading of BDP on the nanoparticles.

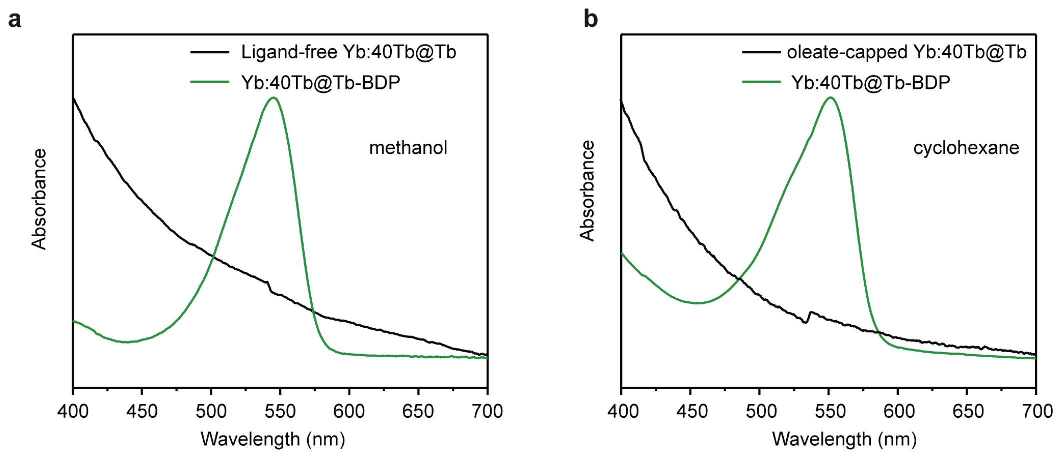

Supplementary Figure 11. a, Absorption spectra of methanol dispersion of ligand-free nanoparticles before and after conjugated with BDP, respectively. **b,** Absorption spectra of cyclohexane dispersion of oleate-capped nanoparticles before and after conjugation with BDP, respectively.

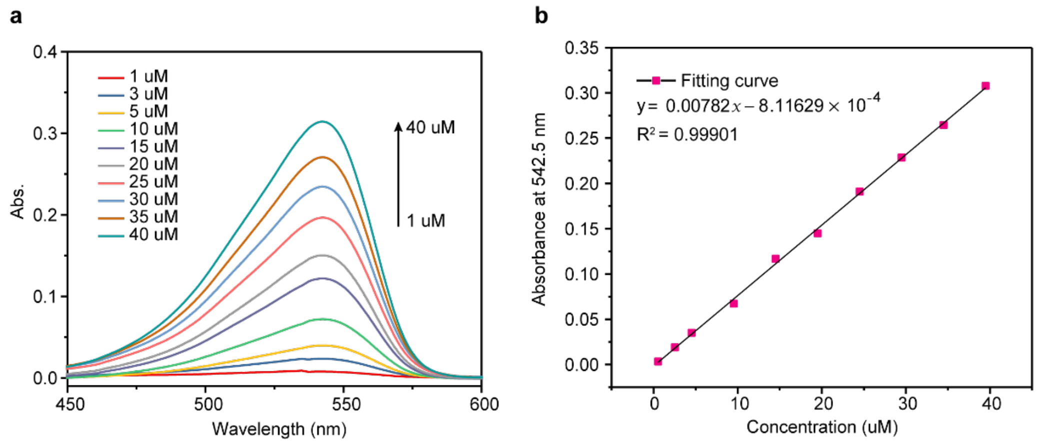

Supplementary Figure 12. a, UV-vis absorption spectra of BDP methanol solution of different concentration. **b,** Plot of the BDP calibration dataset showing the change in absorbance of BDP as a function of concentration.

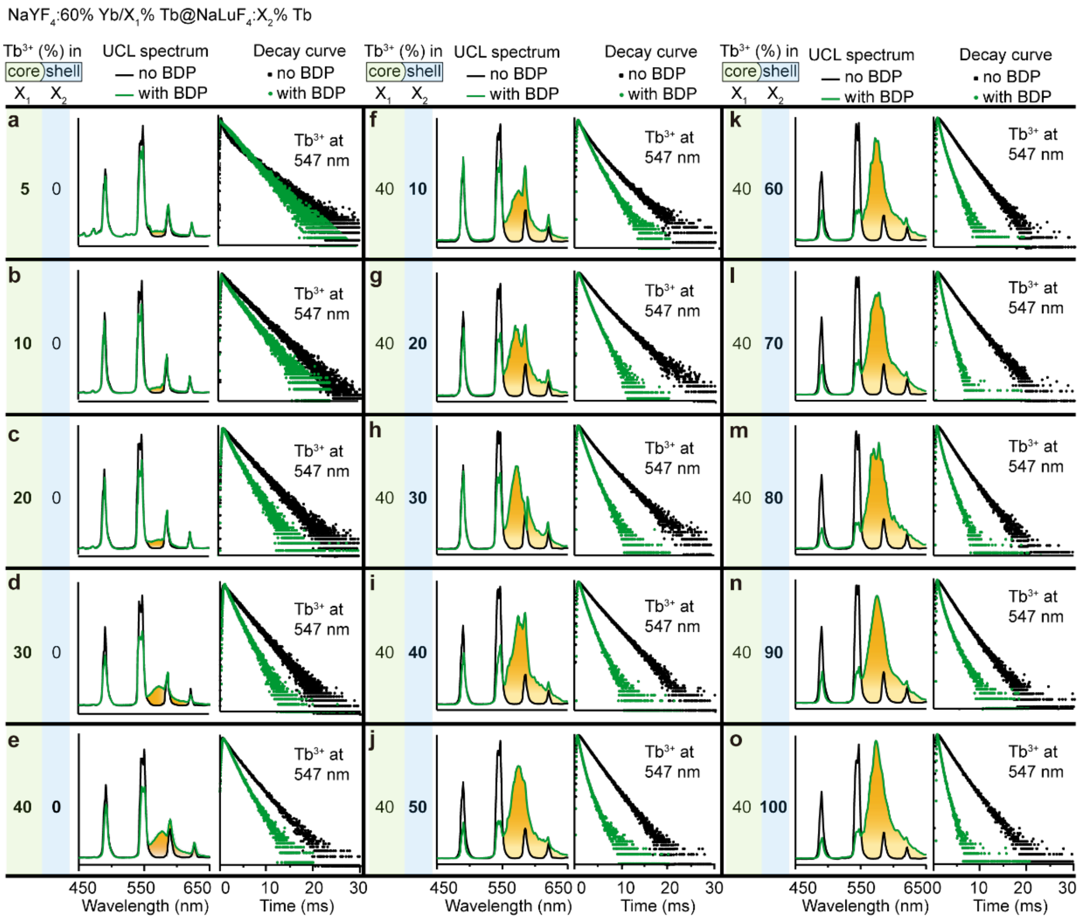

Supplementary Figure 13. Upconversion emission spectra and corresponding luminescence decay curves of **a-e,** $NaYF_4$:Yb,Tb(60, X_1 mol%)@$NaLuF_4$ (X_1=5, 10, 20, 30, 40) and **f-o,** $NaYbF_4$:Tb(40 mol%)@$NaLuF_4$:Tb(X_2 mol%, X_2=10, 20, 30, 40, 50, 60, 70, 80, 90, 100) nanoparticles before and after BDP conjugation, respectively. The sensitized luminescence of BDP molecules is highlighted in orange color. The average loading concentration of BDP is around 34 dyes per particle. Note that the pristine $NaYF_4$:Yb,Tb(60, 5 mol%)@$NaLuF_4$ sample in panel **a** shows a double exponential decay behavior, which is probably due to impurity emission from a trace impurity of Er^{3+} in the sample.

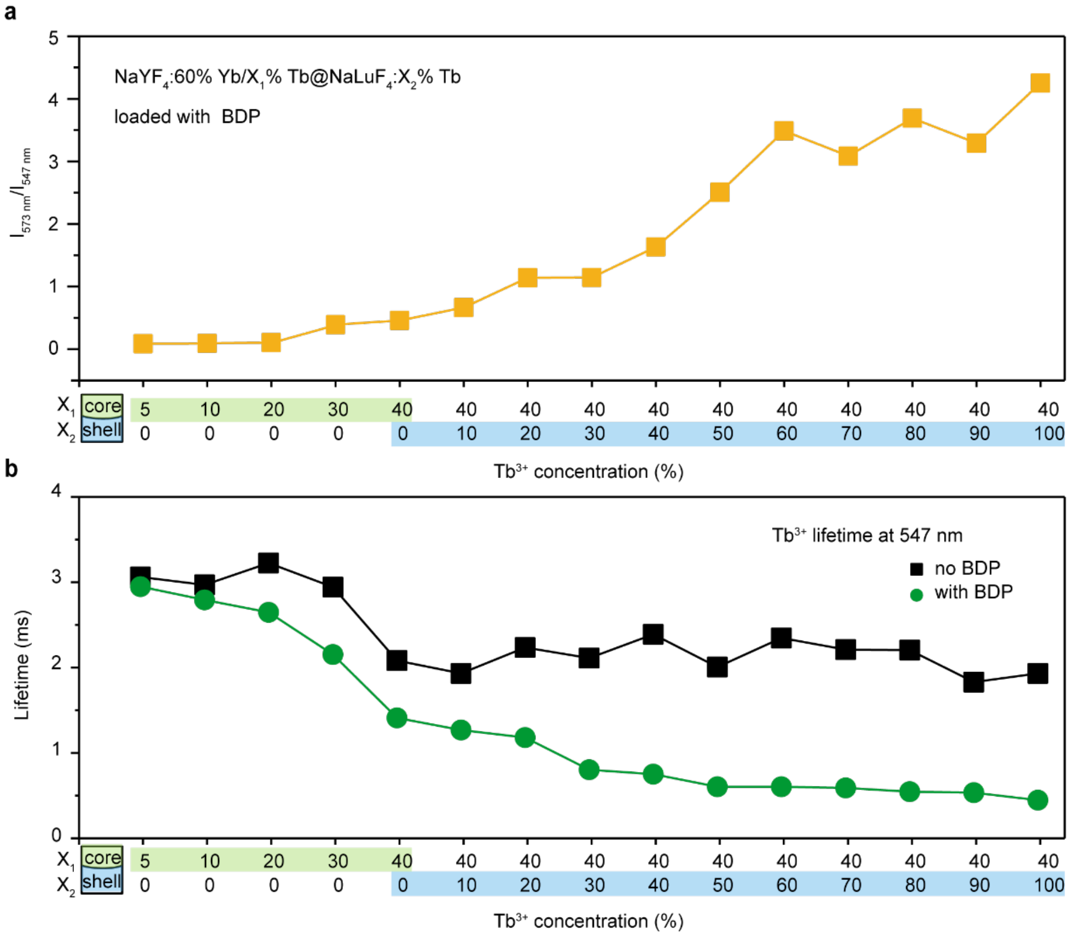

Supplementary Figure 14. a, BDP/Tb^{3+} emission intensity ratios as a function of Tb^{3+} concentration in core-shell nanoparticles. The intensity ratios show an upward trend as Tb^{3+} concentration increases, indicating more efficient nanoparticle to BDP energy transfer at higher Tb^{3+} concentration. **b,** Tb^{3+} concentration-dependent luminescence lifetime of Tb^{3+} recorded at 547 nm. All of the nanoparticles were loaded with an average of ~34 BDP molecules per particle.

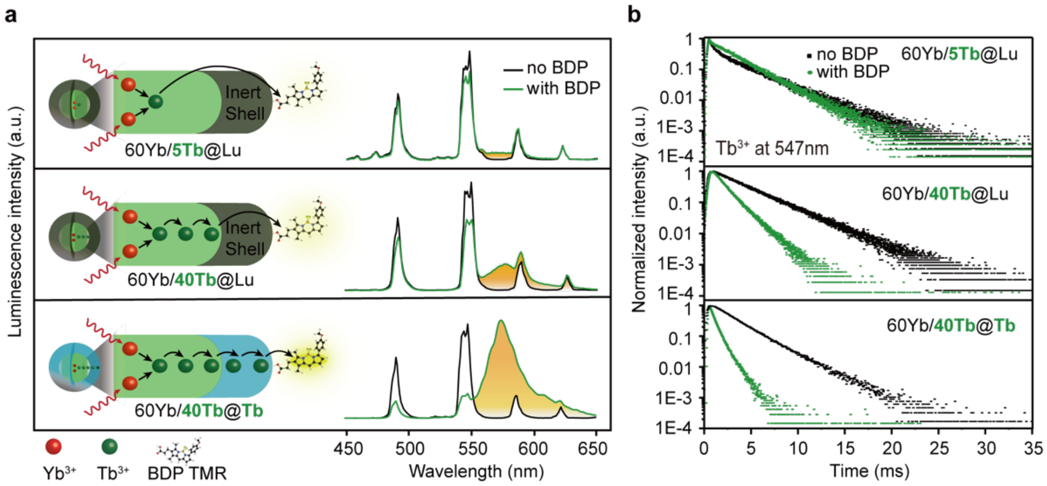

Supplementary Figure 15. a, Upconversion luminescence spectra of $NaYF_4$:Yb/Tb(60,5 mol%)@$NaLuF_4$ (60Yb/5Tb@Lu), $NaYbF_4$:Tb(40 mol%)@$NaLuF_4$ (60Yb/40Tb@Lu), and $NaYbF_4$:Tb(40 mol%)@$NaTbF_4$ (60Yb/40Tb@Tb) nanoparticles recorded before (black lines) and after (green lines) conjugating with BDP molecules. The BDP sensitized emission is highlighted in orange color. The inserted schemes show that the proposed energy transfer processes dominate in different BDP-nanoparticles under investigation. **b,** Corresponding luminescence decay curves of the nanoparticles shown in **a**, as measured at 547 nm before (black dots) and after (green dots) BDP modification.

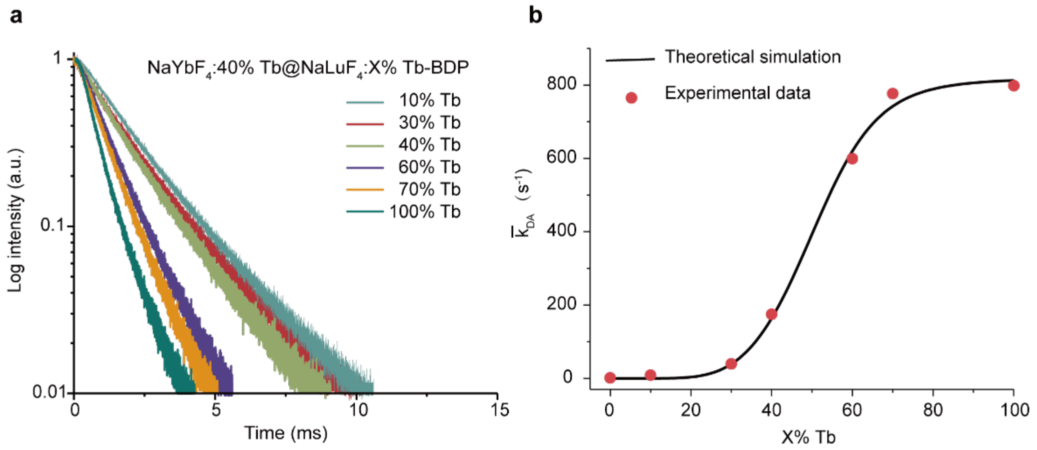

Supplementary Figure 16. Mechanistic investigation of the promoted energy transfer process. **a,** Upconversion luminescence decay curves of Tb^{3+} emission at 547 nm showing the luminescence lifetime changes of Tb^{3+} as a function of Tb^{3+} concentration in the shell level. Note that the average loading number of BDP molecules is ~3.5 per particle. **b,** Concentration dependence of the fit parameter $\bar{k}_{\mathrm{DA}}$ of the Tb^{3+} emission curves. The theoretical simulation was plotted in terms of Eq. S15 and the experimental data (red dots) showing a good fitting between experimental data and the equation.

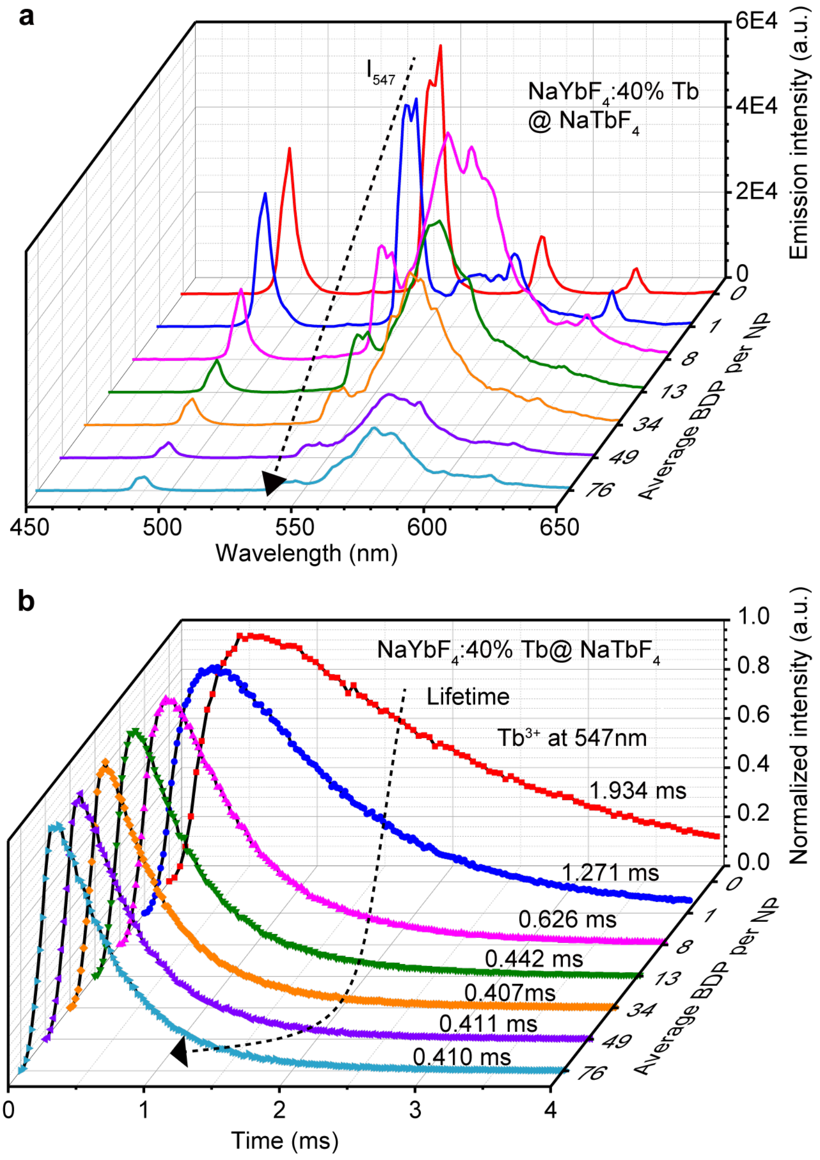

Supplementary Figure 17. Optical characterization of 24-nm $NaYbF_4$:Tb(40 mol%)@$NaTbF_4$ core-shell nanoparticles conjugated with BDP molecules at different concentrations. **a,** Emission spectra of the core-shell nanoparticles conjugated with varied concentrations of BDP. **b,** Corresponding Tb^{3+}-emission (547 nm) decay curves of the samples shown in **a**.

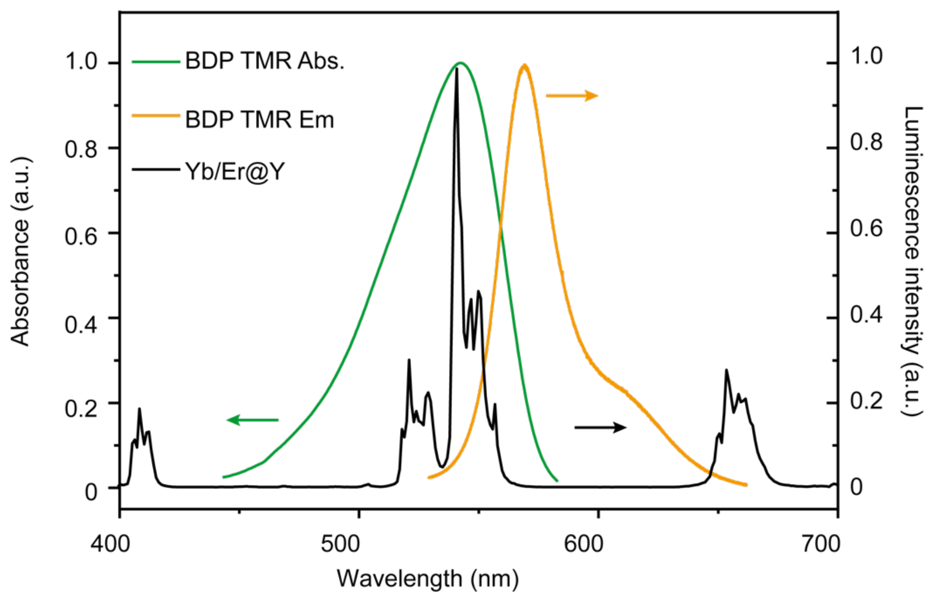

Supplementary Figure 18. Normalized UV-vis absorption (Abs.) and emission (Em.) spectra of BDP molecules and upconversion luminescence spectrum of $NaYF_4$:Yb,Er(18,2 mol%)@$NaYF_4$ (Yb/Er@Y) core-shell nanoparticles. The substantial overlapping between the emission spectrum of Er^{3+} and the absorption spectrum of BDP TMR supports the analysis of the energy transfer mechanism in BDP-conjugated Y/Er@Y systems.

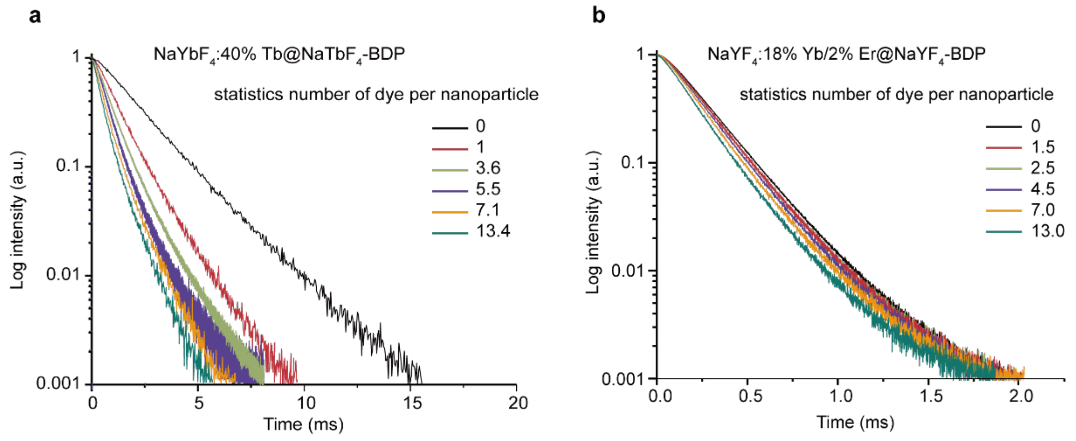

Supplementary Figure 19. Luminescence decay dynamics showing the change in upconversion luminescence lifetime as a function of the number of BDP molecules per particle. **a,** Luminescence decay curves of Tb^{3+} emission at 547 nm recorded for 24 nm $NaYbF_4$:Tb@$NaTbF_4$ nanoparticles. **b,** Luminescence decay curves of Er^{3+} emission at 541 nm, recorded for 24 nm $NaYF_4$:Yb,Er(18,2 mol%)@$NaYF_4$ core-shell nanoparticles.

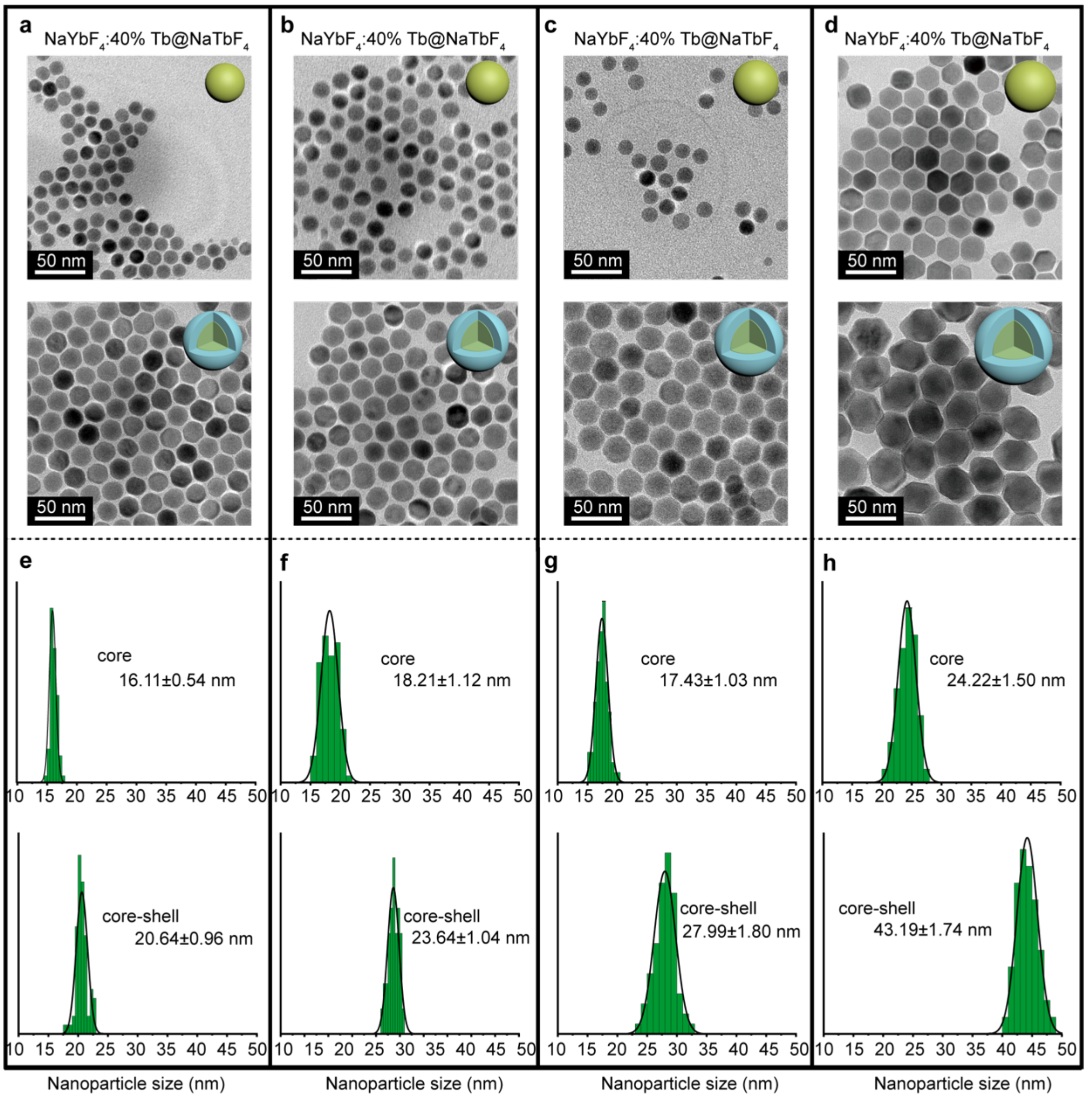

Supplementary Figure 20. **a-d,** TEM images and **e-h,** corresponding size distribution of $NaYbF_4$:Tb core and $NaYbF_4$:Tb@$NaTbF_4$ core-shell nanoparticles with different sizes. The size distributions of the nanocrystals were calculated by counting > 200 particles recorded in TEM images.

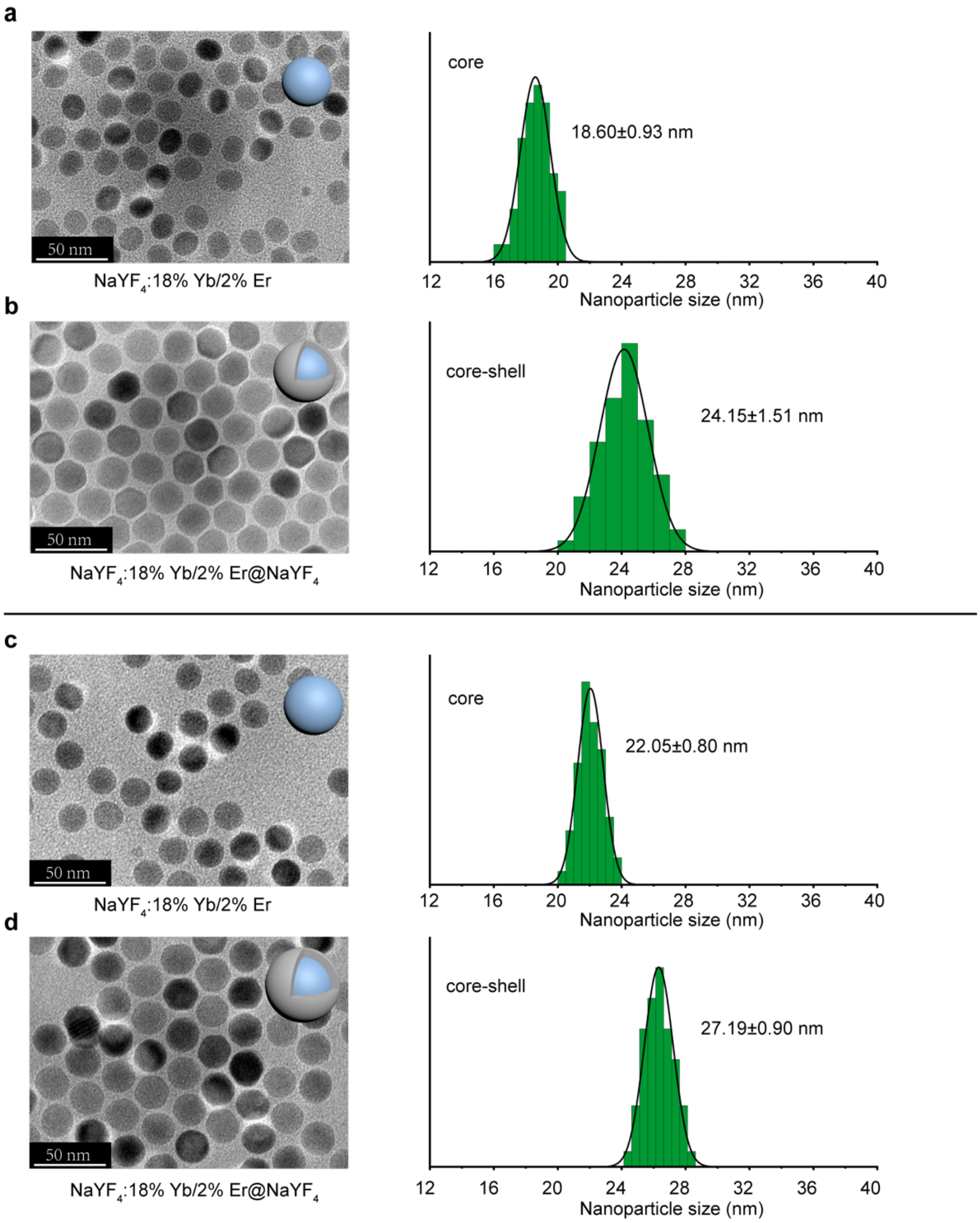

Supplementary Figure 21. TEM images and corresponding size distributions of **a, c** $NaYF_4$:Yb,Er(18,2 mol%) core nanoparticles and **b, d** $NaYF_4$:Yb,Er(18,2 mol%)@$NaYF_4$ core-shell nanoparticles sizes. The size distributions of the nanocrystals were calculated by counting > 200 particles recorded in TEM images.

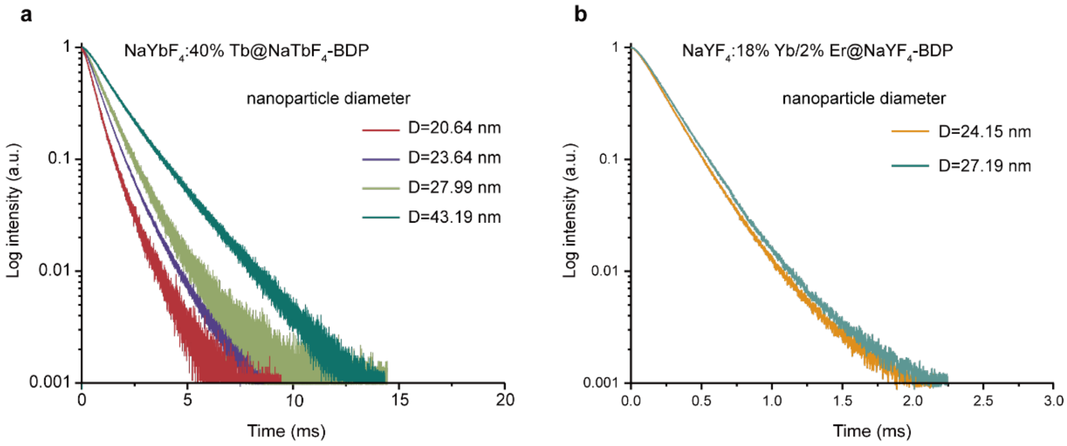

Supplementary Figure 22. Comparison of measured luminescence decay curves of nanoparticles with different sizes loaded with the same amount of BDP molecules. **a,** Upconversion luminescence decay curves of Tb^{3+} emission at 547 nm. **b,** Upconversion luminescence decay curves of Er^{3+} emission at 541 nm. All the nanoparticles have the identical dye loading number of 3 dye molecules per particle.

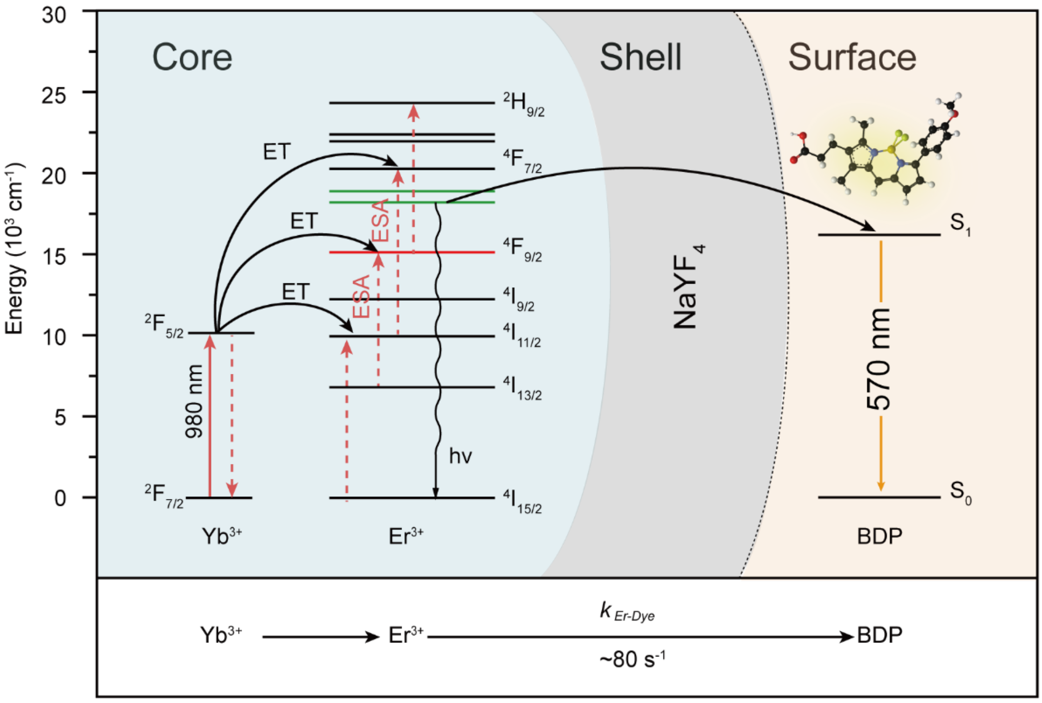

Supplementary Figure 23. Schematic illustration showing the proposed energy transfer processes for BDP-decorated $NaYF_4$:Yb,Er@$NaYF_4$ core-shell nanoparticles.

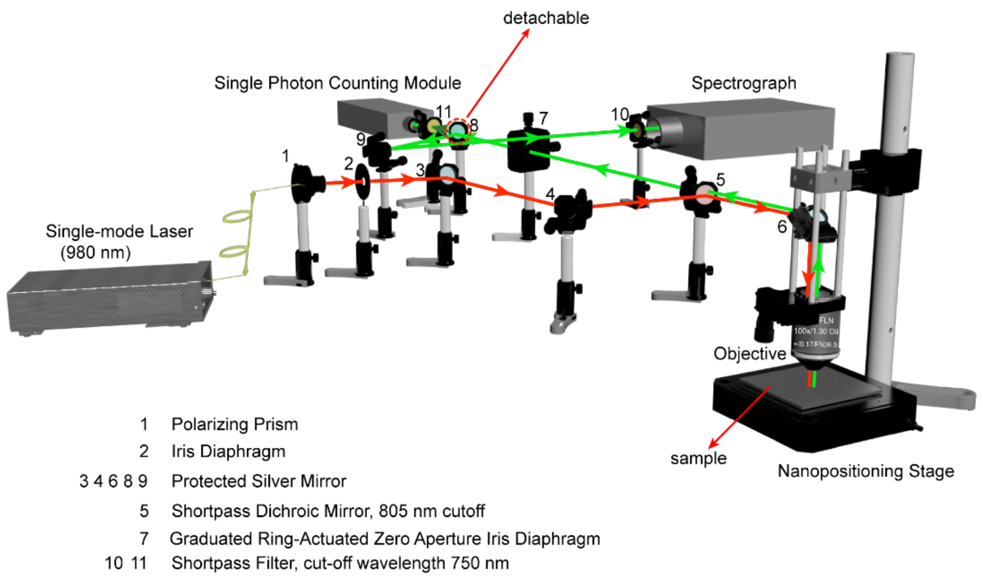

Supplementary Figure 24. Schematic illustration of a homemade confocal scanning microscopy setup used for single-nanoparticle FRET analysis.

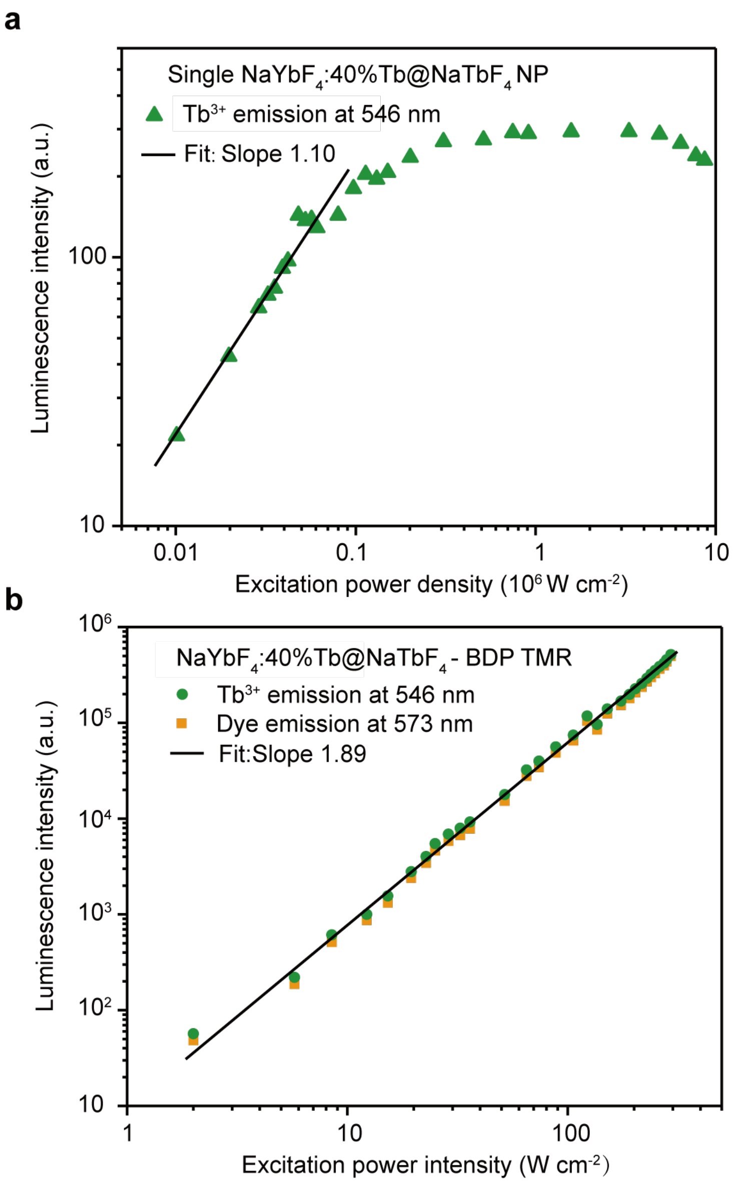

Supplementary Figure 25. Excitation power-dependent upconversion luminescence intensity measured for **a,** a single $NaYbF_4$:Tb(40 mol%)@$NaTbF_4$ core-shell nanoparticle under confocal microscopy, and **b,** BDP-modified $NaYbF_4$:Tb(40 mol%)@$NaTbF_4$ nanoparticles dispersed in methanol.

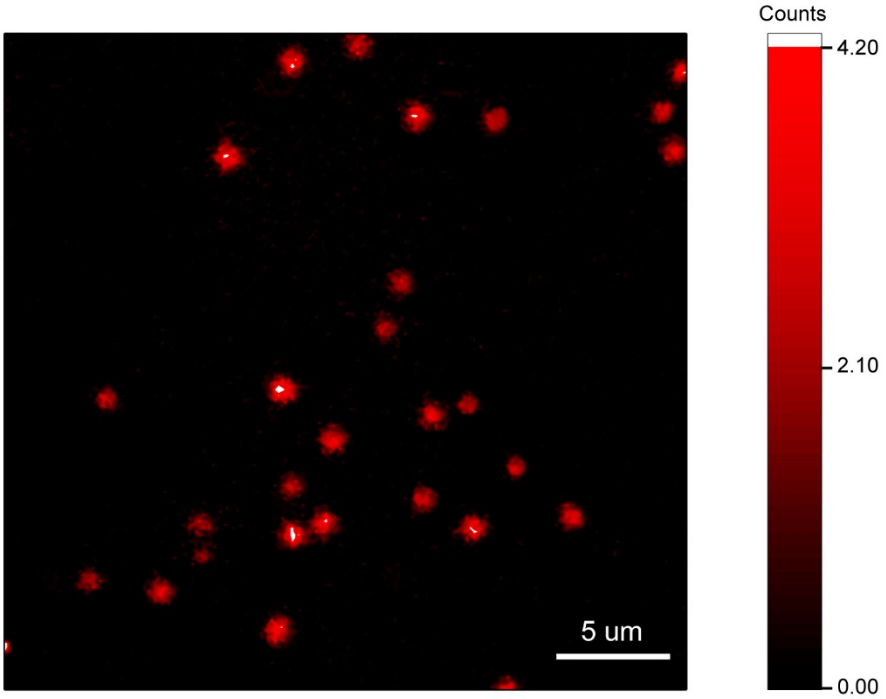

Supplementary Figure 26. A typical confocal upconverted luminescence image of pristine $NaYbF_4$:Tb(40 mol%)@$NaTbF_4$ core-shell nanoparticles under 980 nm excitation.

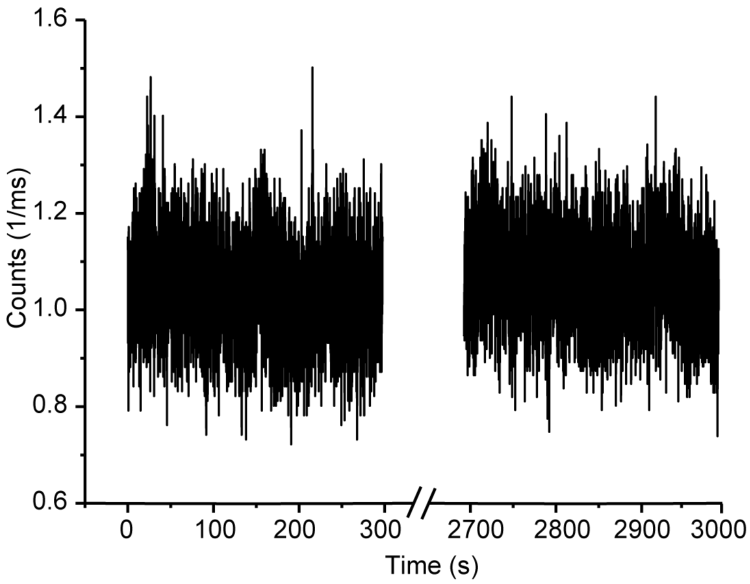

Supplementary Figure 27. Time-course plot of luminescence intensity recorded from a single $NaYbF_4$:Tb(40 mol%)@$NaTbF_4$ nanoparticle, exhibiting exceptional photostability.

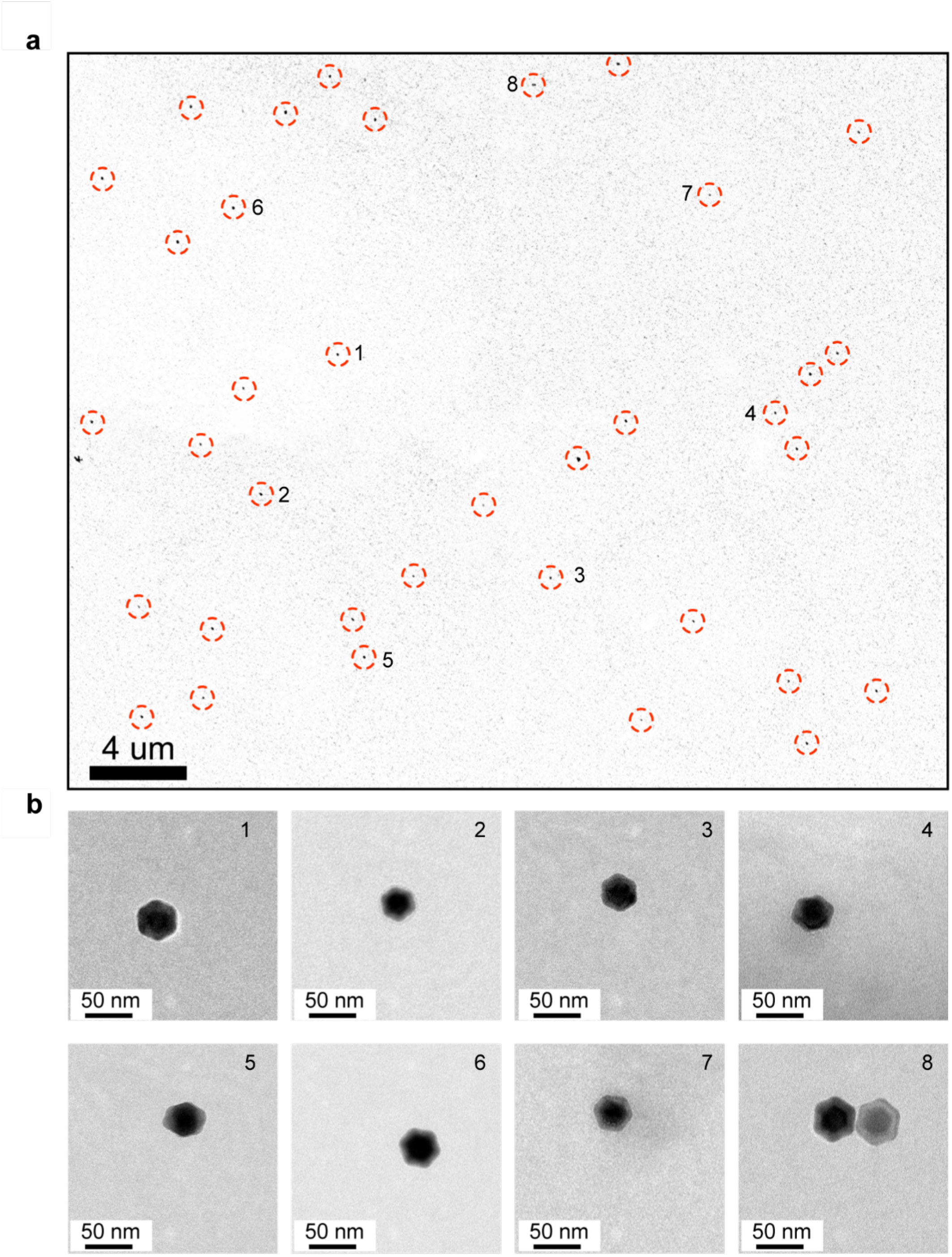

Supplementary Figure 28. TEM characterization of BDP-$NaYbF_4$:Tb(40 mol%)@$NaTbF_4$ nanoparticle conjugates. **a,** Typical TEM image of $NaYbF_4$:Tb(40 mol%)@$NaTbF_4$-BDP at the concentration level used for single-particle optical microscopy imaging. **b,** Zoom-in TEM images showing profiles of single nanoparticles corresponding to small spots shown in **a**.

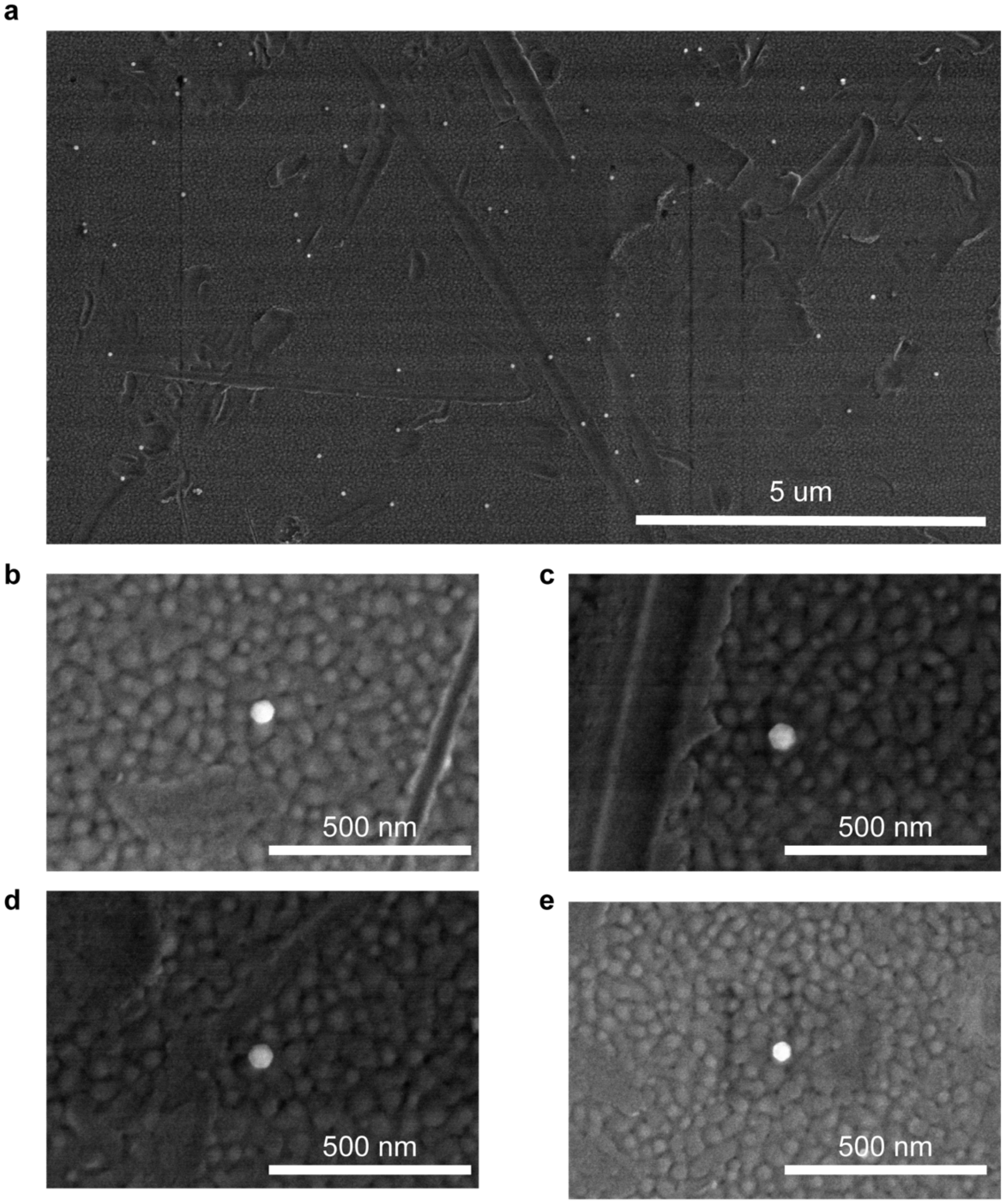

Supplementary Figure 29. SEM characterization of BDP-decorated $NaYbF_4$:Tb@$NaTbF_4$ at the concentration level used for confocal microscopy imaging. **a,** Large area SEM image of BDP-$NaYbF_4$:Tb(40 mol%)@$NaTbF_4$ conjugates. **b-e**, Zoom-in SEM images of random small white spots captured in **a**, indicating high dispersibility of individual nanoparticles.

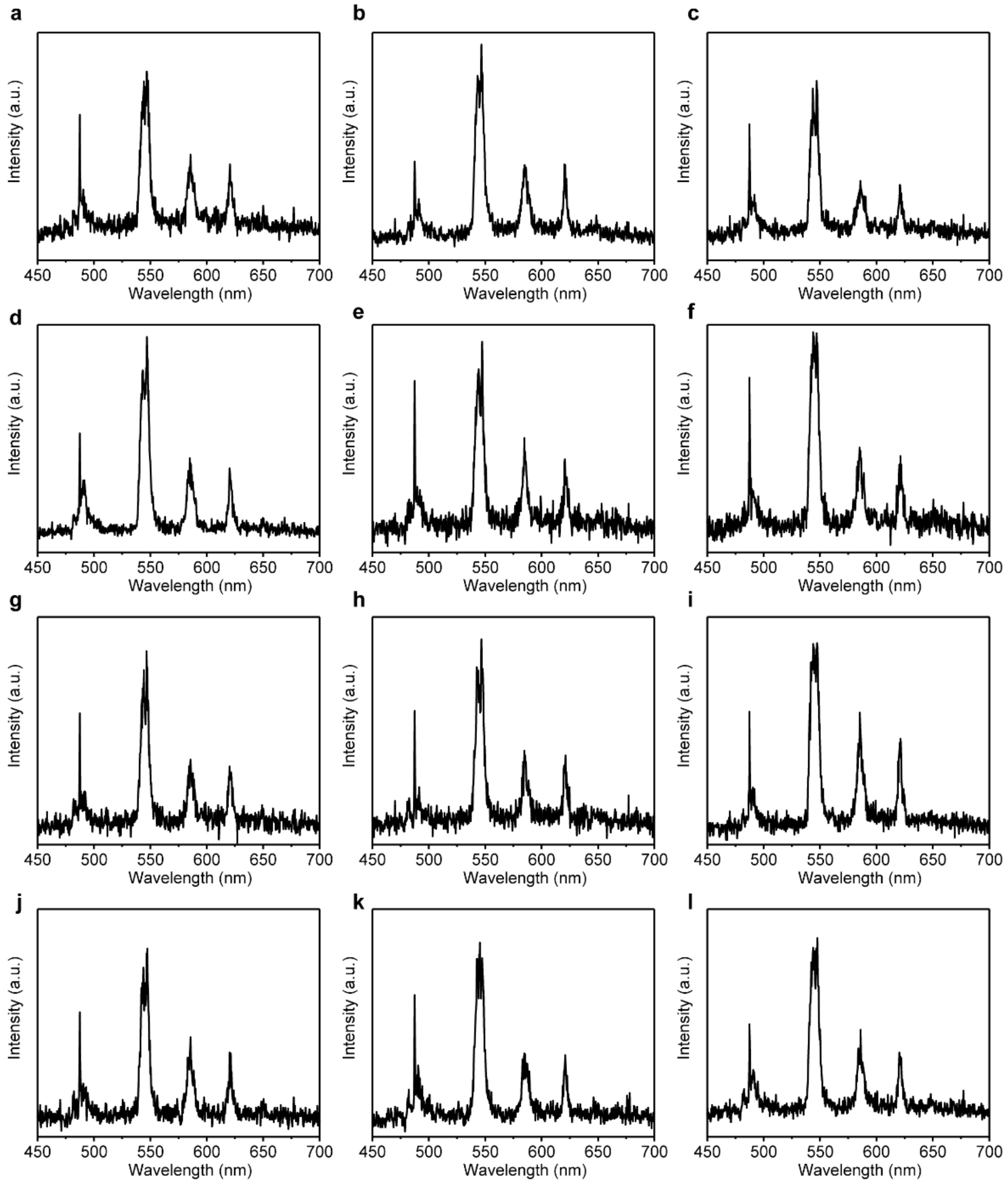

Supplementary Figure 30. Single-particle luminescence spectra of pristine $NaYbF_4$:Tb(40 mol%)@$NaTbF_4$ nanoparticles. The spectra (**a-l**) were recorded from randomly picked luminescence spots by confocal scanning imaging.

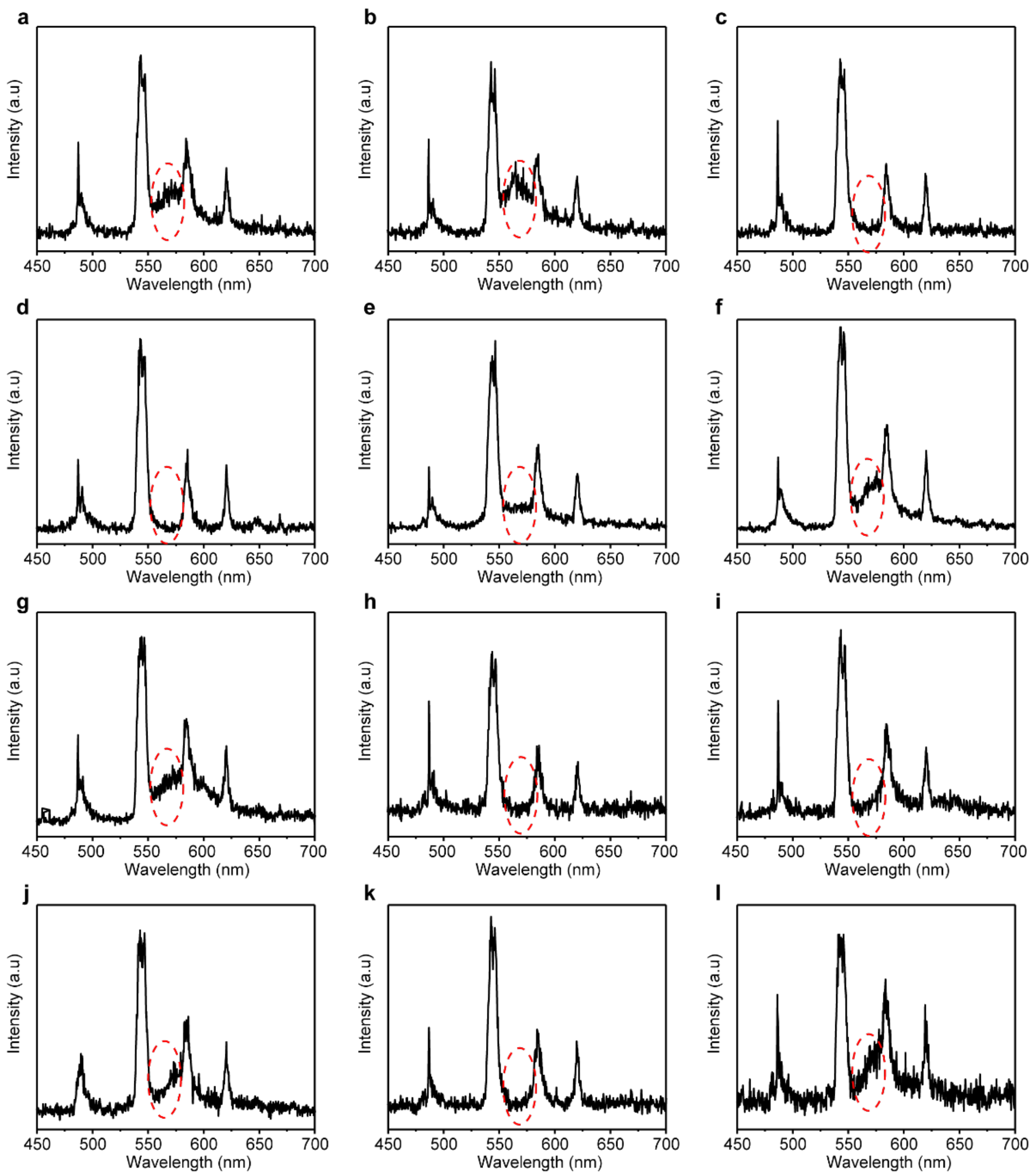

Supplementary Figure 31. Single-particle luminescence spectra of $NaYbF_4$:Tb(40 mol%)@$NaTbF_4$ nanoparticles loaded with an average of 0.9 BDP per nanoparticle. The spectra (**a-l**) were recorded from randomly picked luminescence spots by confocal scanning imaging. The dashed, red circles highlighted the emission spectra of BDP dye molecules.

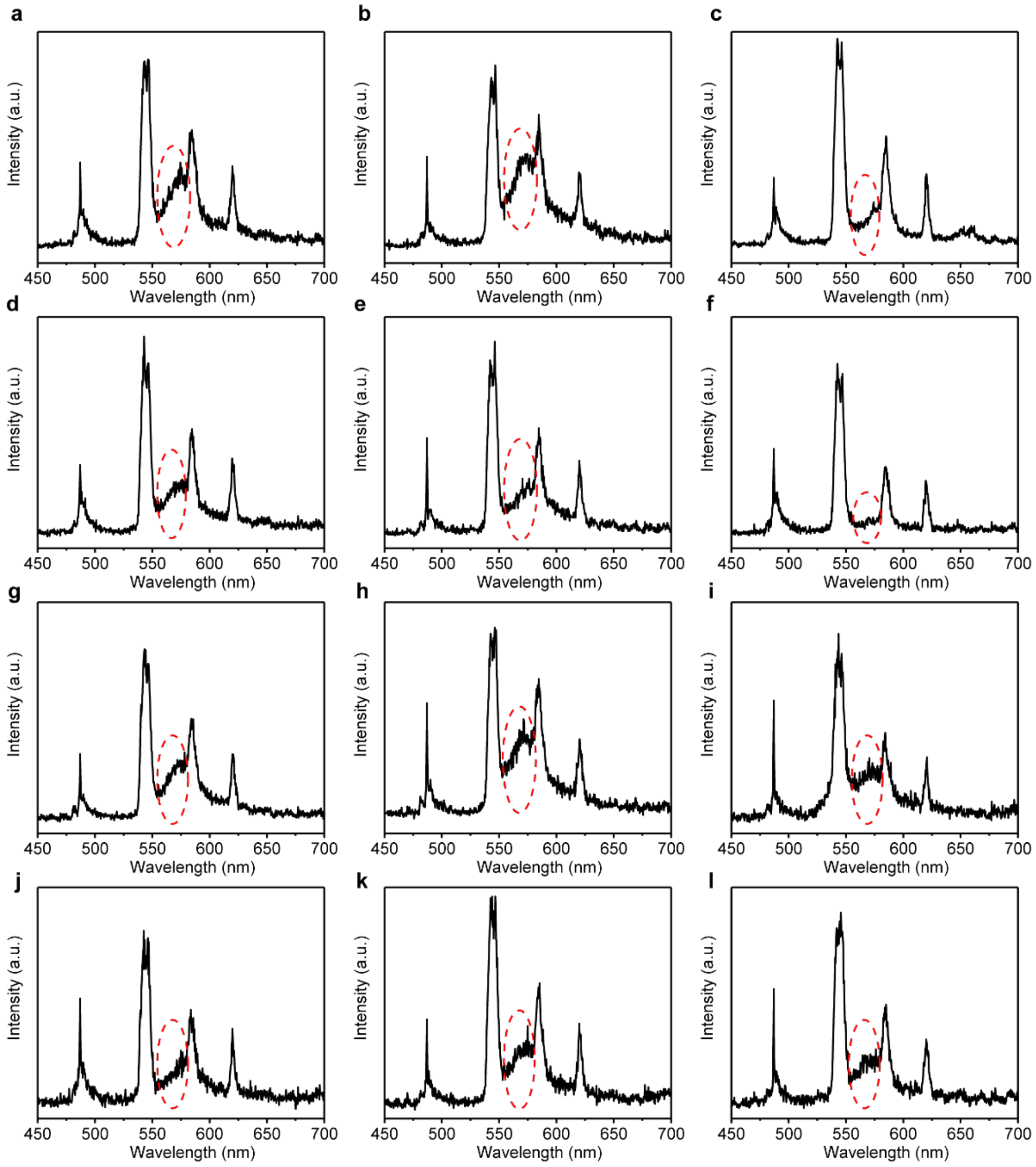

Supplementary Figure 32. Single-particle luminescence spectra of $NaYbF_4$:Tb(40 mol%)@$NaTbF_4$ nanoparticles loaded with an average of 12 BDP per nanoparticle. The spectra **(a-l)** were recorded from randomly picked luminescence spots by confocal scanning imaging. The dashed, red circles highlight the emission spectra of BDP dye molecules.

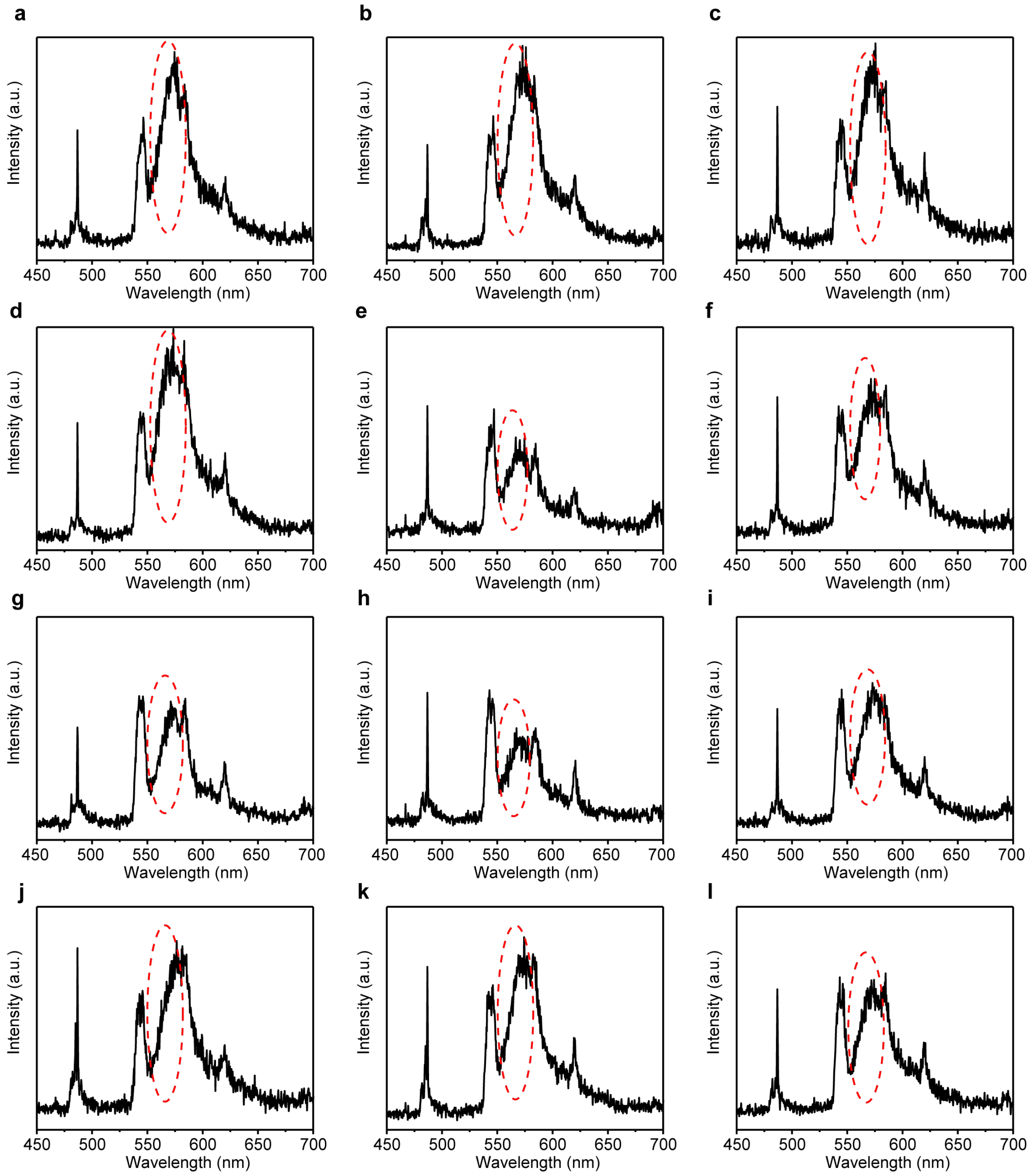

Supplementary Figure 33. Single-particle luminescence spectra of $NaYbF_4$:Tb(40 mol%)@$NaTbF_4$ nanoparticles loaded with average 52 BDP per nanoparticle. The spectra (**a-l**) were recorded from randomly picked luminescence spots by confocal scanning imaging. The dashed, red circles highlight the emission spectra of BDP dye molecules.

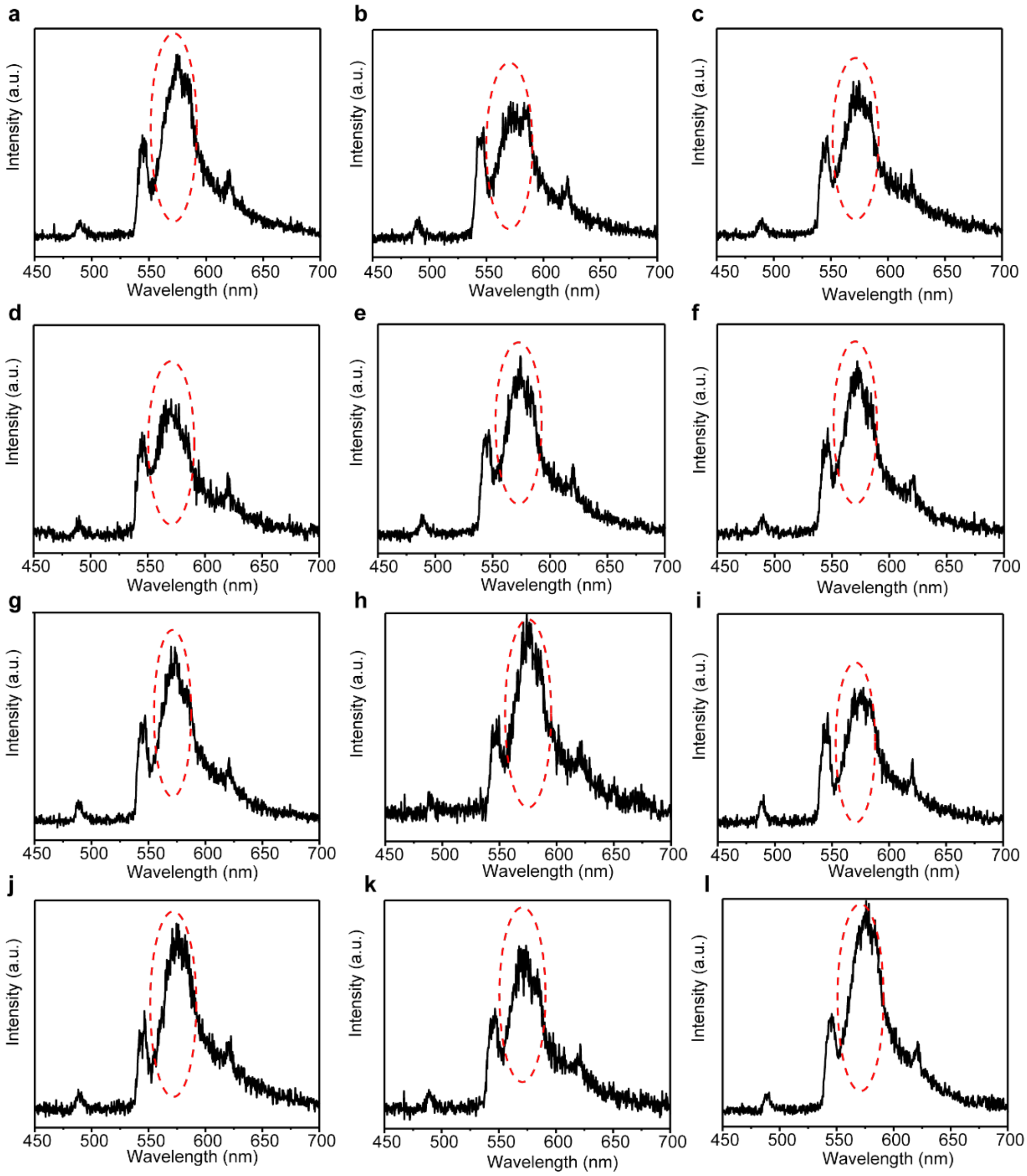

Supplementary Figure 34. Single-particle luminescence spectra of $NaYbF_4$:Tb(40 mol%)@$NaTbF_4$ nanoparticles loaded with average 92 BDP per nanoparticle. The spectra **(a-l)** were recorded from randomly picked luminescence spots by confocal scanning imaging. The dashed, red circles highlight the emission spectra of BDP dye molecules.

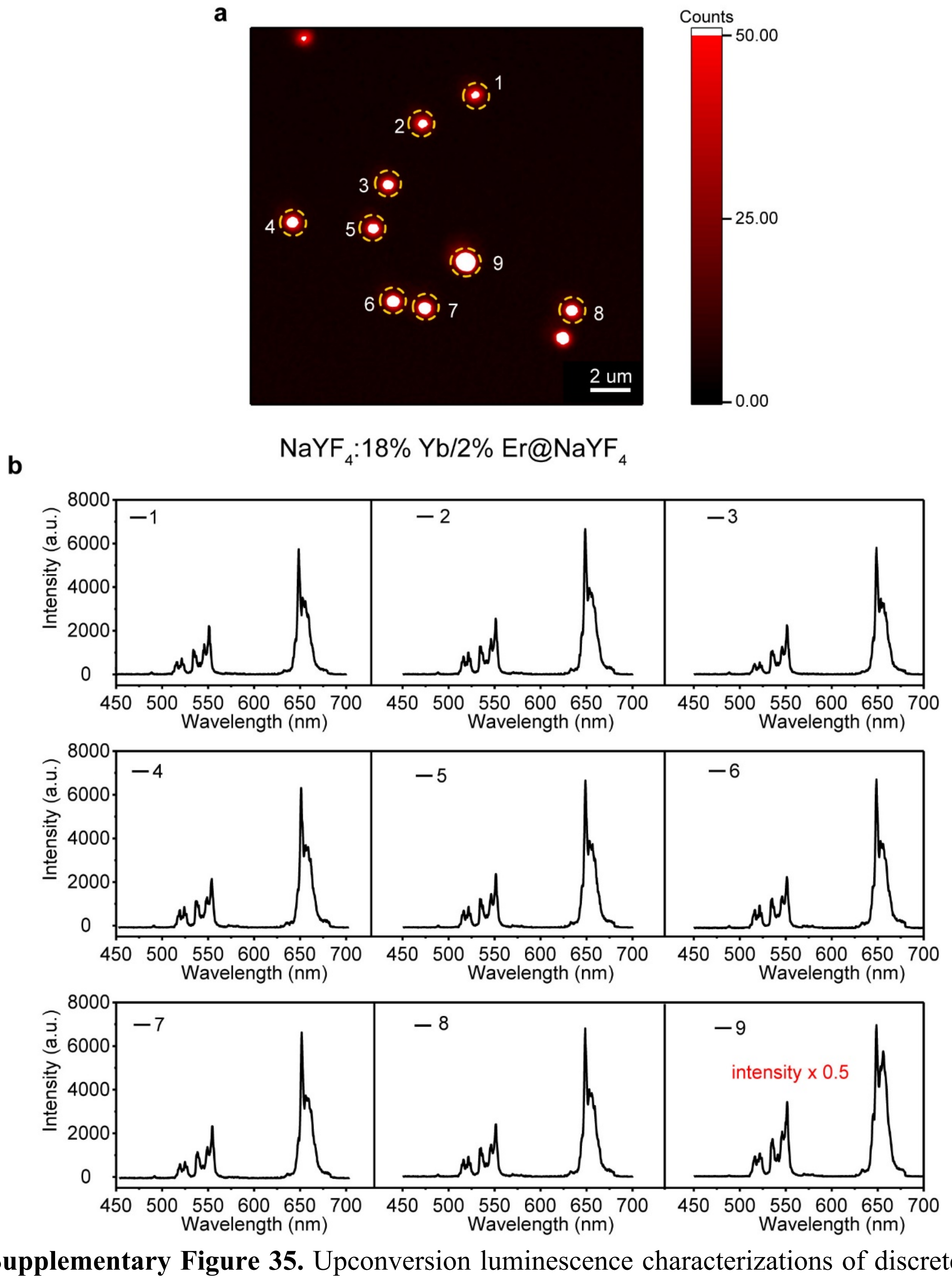

Supplementary Figure 35. Upconversion luminescence characterizations of discrete $NaYF_4$:Yb,Er(18,2 mol%)@$NaYF_4$ nanoparticles. **a,** Confocal luminescence imaging of a nanoparticle-dispersed sample specimen under 980 nm excitation. **b,** Upconversion luminescence spectra of 9 individual nanoparticles, corresponding to luminescent spots shown in **a**. Note that spots 1 to 8 show similar intensities both in the confocal scanning image and the emission spectra. The intensity of spot 9 is two times higher than other spots, indicating the existence of a particle dimer at spot 9.

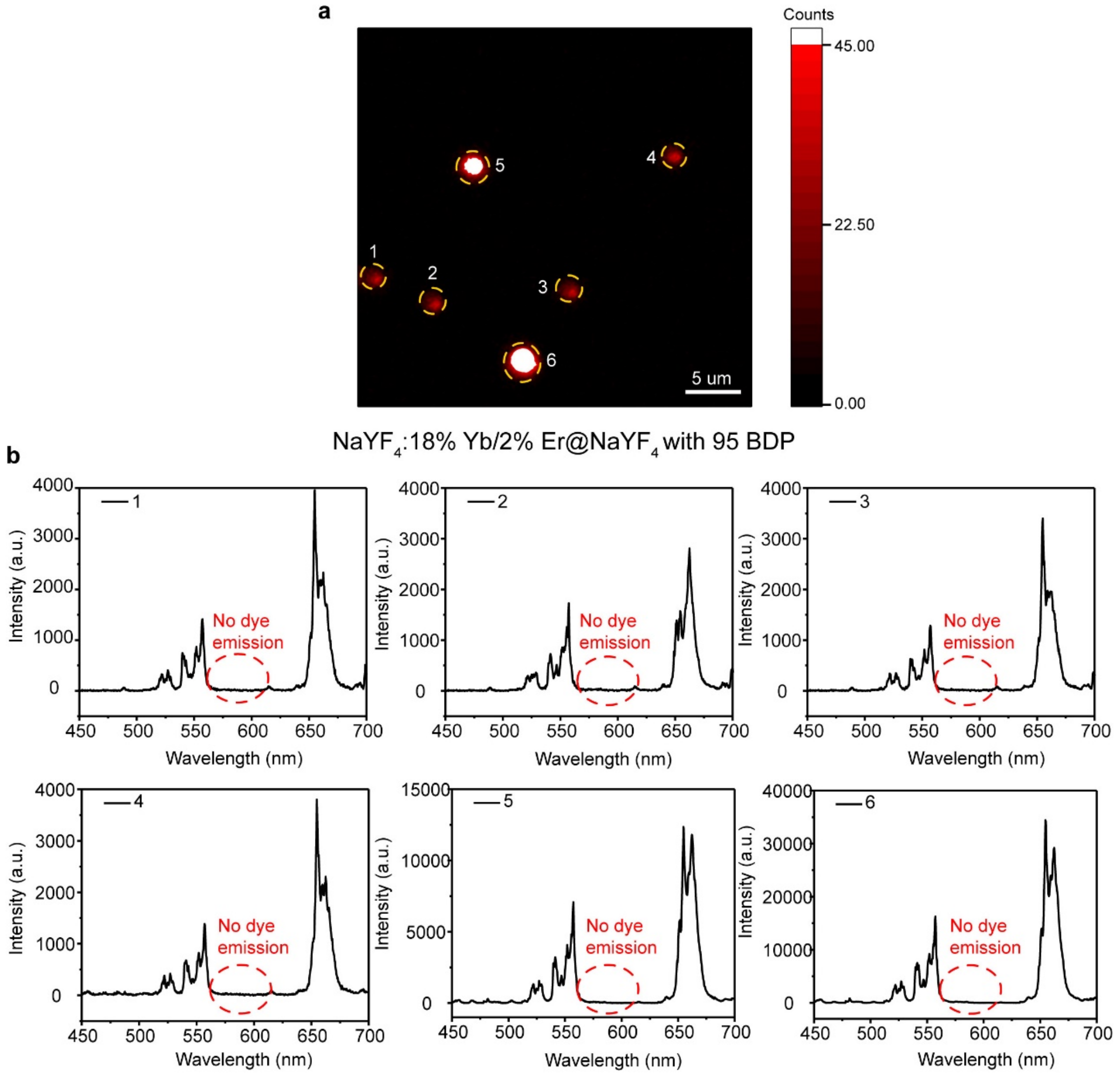

Supplementary Figure 36. Microscopy imaging of discrete BDP-decorated $NaYF_4$:Yb,Er(18,2 mol%)@$NaYF_4$ nanoparticles. **a,** Confocal upconversion luminescence imaging of the sample under 980 nm excitation. **b,** Corresponding upconversion emission spectra of 6 BDP-nanoparticle conjugates shown in **a**. Note that no sensitized dye emission is detected in these nanoparticles, albeit the high loading number of dye molecules (95 BDP per particle).

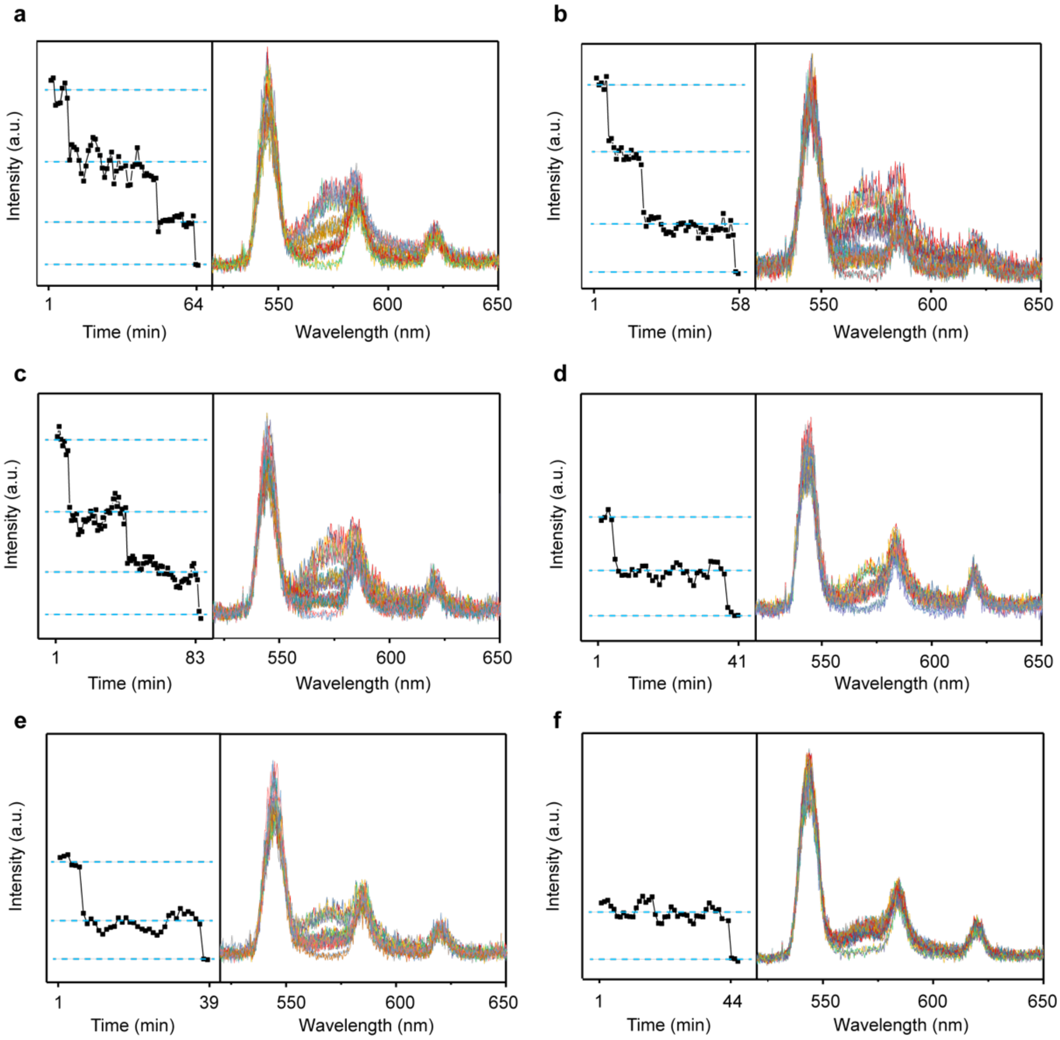

Supplementary Figure 37. Tracking single-molecule photobleaching by upconversion nanoprobes**.** Time-correlated, single-particle emission spectra of BDP-decorated $NaYbF_4$:Tb(40 mol%)@$NaTbF_4$ nanoparticles (~0.9 BDP per particle) recorded from individual nanoparticles, showing distinguishable stepwise photobleaching of BDP emission containing (**a-c**) 3 BDP molecules, (**d-e**) 2 BDP molecules and (**f**) 1 BDP molecule.

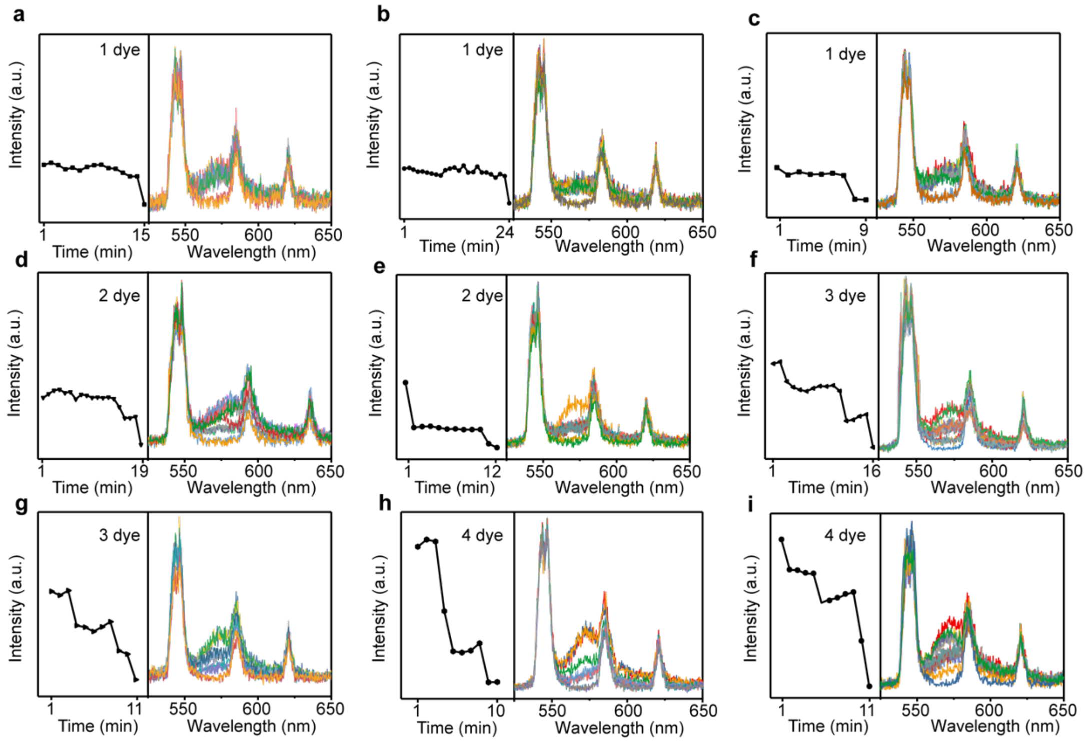

Supplementary Figure 38. Tracking single-molecule photobleaching by upconversion nanoprobes**.** Time-correlated single-particle emission spectra of BDP-decorated $NaYbF_4$:Tb(40 mol%)@$NaTbF_4$ nanoparticles (~0.9 BDP per particle) from 9 randomly picked nanoparticles, showing distinguishable stepwise photobleaching from individual nanoparticles containing (**a-c**) 1 BDP molecule, (**d-e**) 2 BDP molecules, (**f-g**) 3 BDP molecules, and (**h-i**) 4 BDP molecules. Note that 450 nM H_2O_2 was added to the stock solution of BDP-decorated $NaYbF_4$:Tb@$NaTbF_4$ nanoparticles before drop-casting the sample.

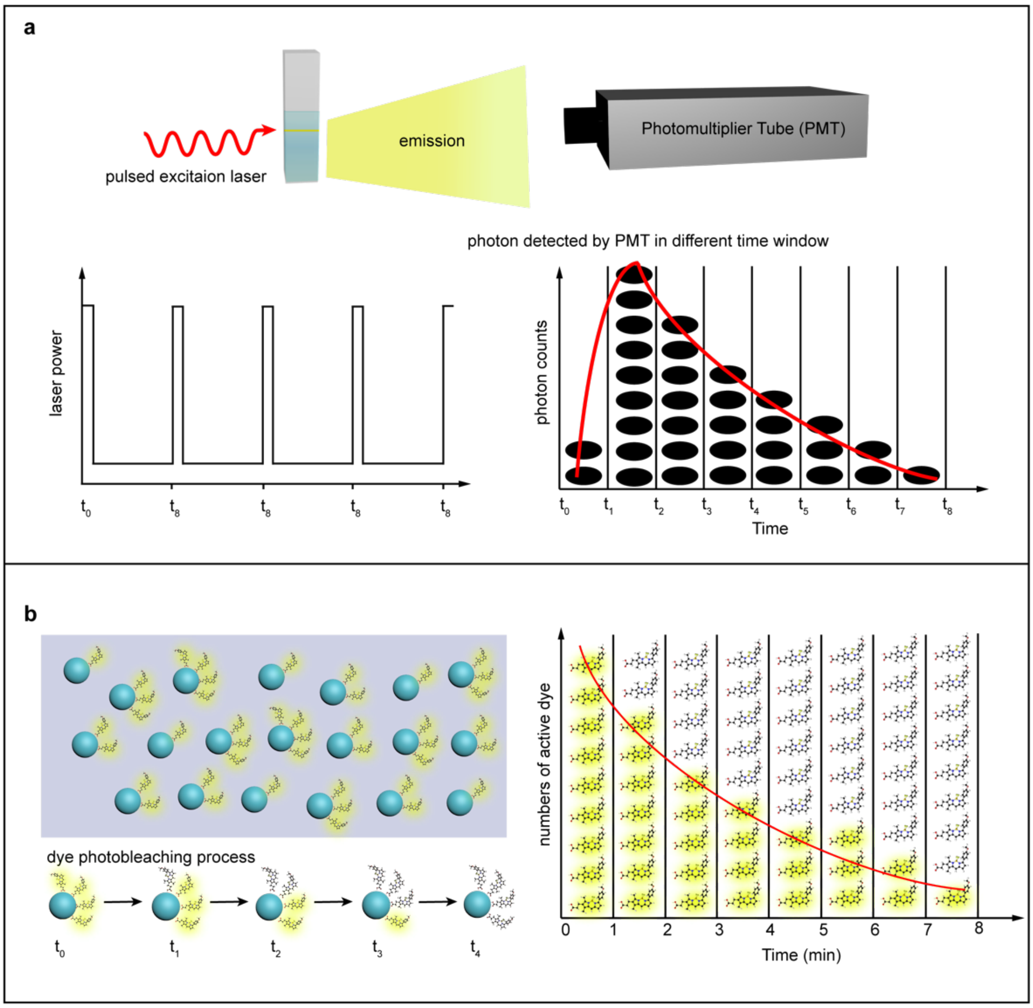

Supplementary Figure 39. a, Schematic illustration of time-correlated single-photon counting (TCSPC). The photomultiplier is used to detect the emission photons from the sample excited by a pulse laser. Each cycle (t_0-t_8) can be divided into multiple time windows. Note that no more than one single photon event per cycle is detected. **b,** Schematic diagram shwoing the photobleaching kinetic analysis of dye molecules by a single-molecule counting method.

Supplementary Note 3

We adopted a single molecule counting method inspired by TCSPC to explore the reaction kinetics of the photobleaching process. TCSPC is a well-established technique for fluorescence lifetime measurements[12]. In TCSPC, molecular fluorescence is excited by a sequence of short laser pulses. The photons emitted from the molecules are determined by a photomultiplier tube (PMT). By setting up specific "time bins" channels, the detected photons arrived at the same time interval after a pulse excitation are counted in one channel. With a high repetition rate of the excitation source, millions of sequences are measured. This results in a histogram of photon counts in several serial

time channels, and the histogram represents the dynamic fluorescence change as a function of time (Supplementary Figure 39a).

In our experiments, we first measured the single-molecule photobleaching of individual nanoparticles by examining the stepwise quenching of BDP luminescence under continuous laser irradiation, as illustrated in Supplementary Figure 37 and 38. For every one minute, we captured a luminescence spectrum to monitor the intensity dropping of BDP. We recorded the events of the stepwise quenching for an individual nanoparticle until the emission of BDP was completely quenched. The measurement cycle was repeated on several randomly picked single nanoparticles until enough number of quenching events were recorded. By gathering all of the quenching events from a large number of individual measurements, we can count the number of survived dye molecules versus irradiation time. This allows us to generate a time-dependent histogram reflecting the reaction kinetics of single-molecule photobleaching on upconversion nanoparticles (Supplementary Figure 39b).

Supplementary Table 1. Characterization of energy transfer efficiency from nanoparticles to BDP molecules as a function of Tb^{3+} concentration.

Tb^{3+} core X_1 %	in the shell X_2 %	Lifetime of Tb^{3+} at 547 nm without BDP (ms) τ_D	Lifetime of Tb^{3+} at 547 nm with BDP (ms) τ_{DA}	Energy transfer effciency	I_{573}/I_{547}
5	0	3.067	2.954	3.7%	0.086
10	0	3.074	2.798	9.0%	0.092
20	0	3.232	2.652	17.9%	0.105
30	0	2.948	2.156	26.9%	0.390
40	0	2.085	1.411	32.3%	0.458
40	10	1.934	1.269	34.4%	0.664
40	20	2.239	1.181	47.3%	1.142
40	30	2.114	0.803	62.0%	1.145
40	40	2.394	0.750	68.7%	1.634
40	50	2.009	0.602	70.0%	2.507
40	60	2.351	0.602	74.4%	3.489
40	70	2.214	0.589	73.4%	3.086
40	80	2.208	0.544	75.4%	3.691
40	90	1.832	0.533	71.0%	3.291
40	100	1.934	0.445	77.0%	4.252

*The BDP loading concentration is ~34 dyes per nanoparticle and the energy transfer efficiency (*Eff*) was calculated by the equation $Eff = 1 - \frac{\tau_{DA}}{\tau_D}$.

Supplementary Table 2. The donor density (N_D) and the experimental $\overline{k}_{DA}$ for Tb^{3+}-doped nanoparticles

X mol% Tb	10	30	40	60	70	100
N_D ($m^{-3}/10^{27}$)	1.43	4.23	5.6	8.27	10.88	13.4
$\overline{k}_{DA}$ (s^{-1})	7.4	38.1	173.4	598.1	775	797

Supplementary Table 3. Determination of loading concentration of BDP on core-shell nanoparticles with a diameter of ~24 nm.

Samples	Absorbance intensity at 542.5 nm	BDP TMR loading concentration (uM)	Average number of BDP TMR per nanoparticle
$NaYbF_4$:40% Tb@$NaTbF_4$ (1.4 uM)	0.0044	0.672	0.5
	0.0097	1.349	1.0
	0.0785	10.922	7.8
	0.1461	18.785	13.4
	0.3700	47.422	33.9
	0.5402	69.183	49.4
	0.8276	105.937	75.7

Supplementary Table 4. Characterization of energy transfer efficiency as a function of dye loading concentration.

Samples	Average number of dye per nanoparticle	Average $\overline{\tau}_{DA}$ (ms)	Average energy transfer efficiency
$NaYbF_4$:Tb(40 mol%)@$NaTbF_4$	1.0	1.449± 0.158	25.1%± 8.2%
	3.6	0.891± 0.085	53.9%± 4.4%
τ_D=1.934 ms	5.4	0.724± 0.047	62.6%± 2.5%
(i-SET)	7.1	0.685± 0.050	64.6%± 2.6%
	13.4	0.501± 0.052	74.1%± 2.7%
$NaYF_4$:Yb,Er(18,2 mol%)@$NaYF_4$	1.7	0.208 ± 0.002	3.7% ± 0.8%
	2.6	0.206 ± 0.001	4.5% ± 0.3%
τ_D=0.216 ms	4.5	0.202 ± 0.004	6.5% ± 1.7%
(Direct ET)	6.9	0.193 ± 0.003	10.8% ±1.4%
	12.9	0.181 ± 0.010	16.4% ±4.6%

$\overline{\tau}_{DA}$ is calculated by three different tests of the corresponding sample.

Supplementary Table 5. The measured energy transfer efficiency from nanoparticle with different sizes.

Samples	Diameter of nanoparticle (nm)		Average $\overline{\tau}_{DA}$ (ms)	Energy transfer efficiency
	core	core-shell		
$NaYbF_4$:Tb(40 mol%)@$NaTbF_4$	16.11±0.54	20.64±0.96	0.628± 0.019	67.5%± 0.9%
μ=4	18.21±1.12	23.64±1.04	0.789± 0.032	60.9% ± 1.7%
τ_D=1.934 ms	17.43±1.03	27.99±1.80	1.174± 0.223	34.7%± 11.5%
(i-SET)	24.22±1.50	43.19±1.74	1.491± 0.067	22.9%± 3.4%
$NaYF_4$:Yb,Er(18,2 mol%)@$NaYF_4$ μ=4	18.60±0.93	24.15±1.51	0.209± 0.005	3.3% ± 2.3%
τ_D=0.216 ms (Direct ET)	22.05±0.80	27.19±0.90	0.213 ± 0.002	1.5% ± 0.8%

$\overline{\tau}_{DA}$ is calculated by three different tests of the corresponding sample.

Supplementary References

1. Wang, F., Deng, R. & Liu.X. Preparation of core-shell $NaGdF_4$ nanoparticles doped with luminescent lanthanide ions to be used as upconversion-based probes. *Nat.Protoc.* **9**, 1634–1644 (2014).
2. Zhou, B. *et al.* Photon upconversion through Tb^{3+}-mediated interfacial energy transfer. *Adv. Mater.* **27**, 6208–6212 (2015).
3. Voss, B. & Haase, M. Intrinsic focusing of the particle size distribution in colloids containing nanocrystals of two different crystal phases. *ACS Nano*. **7**, 11242–11254 (2013).
4. Roberts, J. E. Lanthanum and neodymium salts of trifluoroacetic acid. *J. Am. Chem. Soc.* **83**, 1087–1088 (1961).
5. Mai, H.X. *et al*. Size- and phase-controlled synthesis of monodisperse $nayf_4$:yb,er nanocrystals from a unique delayed nucleation pathway monitored with upconversion spectroscopy. *J. Phys. Chem. C.* **111**, 13730–13739 (2007).
6. Mai, H.X. *et al*. High-quality sodium rare-earth fluoride nanocrystals: controlled synthesis and optical properties. *J. Am. Chem. Soc.* **128**, 6426–6436 (2006).
7. Bogdan,N. *et al*. Synthesis of ligand-free colloidally stable water dispersible brightly luminescent lanthanide-doped upconverting nanoparticles. *Nano Lett*. **11**, 835–840 (2011).
8. Santos, M. B. *et al.* On the distribution of the nearest neighbor. *Am. J. Phys*. **54**, 1139–1141 (1986).
9. Almgren, M. & Löfroth, J. E. Effects of polydispersity on fluorescence quenching. in micelles. *J. Chem. Phys*. **76**, 2734–2743 (1982).
10. Burshteĭn, A. I. Concentration quenching of noncoherent excitation in solutions, *Sov. Phys. Usp.* **27,** 579-606 (1984).
11. Soos, Z. G. & Powell, R. C. Generalized random-walk model for singlet-exciton energy transfer. *Phys. Rev. B.* **6**, 4035–4046 (1972).
12. Hirvonen, L. M. & Suhling, K. Wide-field TCSPC: methods and applications. *Meas. Sci. Technol.* **28**, 012003 (2017).
